# Cytotoxic Sesquiterpenoids from *Ammoides atlantica* Aerial Parts

**DOI:** 10.1021/acs.jnatprod.1c01211

**Published:** 2022-02-23

**Authors:** Sihem Boudermine, Valentina Parisi, Redouane Lemoui, Tarek Boudiar, Maria Giovanna Chini, Silvia Franceschelli, Michela Pecoraro, Maria Pascale, Giuseppe Bifulco, Alessandra Braca, Nunziatina De Tommasi, Marinella De Leo

**Affiliations:** † Département de Chimie, Université de Constantine 1, Constantine, 25000, Algeria; ‡ Département de Chimie, Université de 20 Aout 1955, Skikda, 21000, Algeria; § Dipartimento di Farmacia, Università degli Studi di Salerno, 84084 Fisciano (SA), Italy; ⊥ Biotechnology Research Center, Constantine, 25000, Algeria; ∥ Dipartimento di Bioscienze e Territorio, 86090 Pesche (IS), Italy; ¶ Dipartimento di Farmacia, Università di Pisa, 56126 Pisa, Italy; ○ CISUP, Centro per l’Integrazione della Strumentazione Scientifica, Università di Pisa, 56126 Pisa, Italy

## Abstract

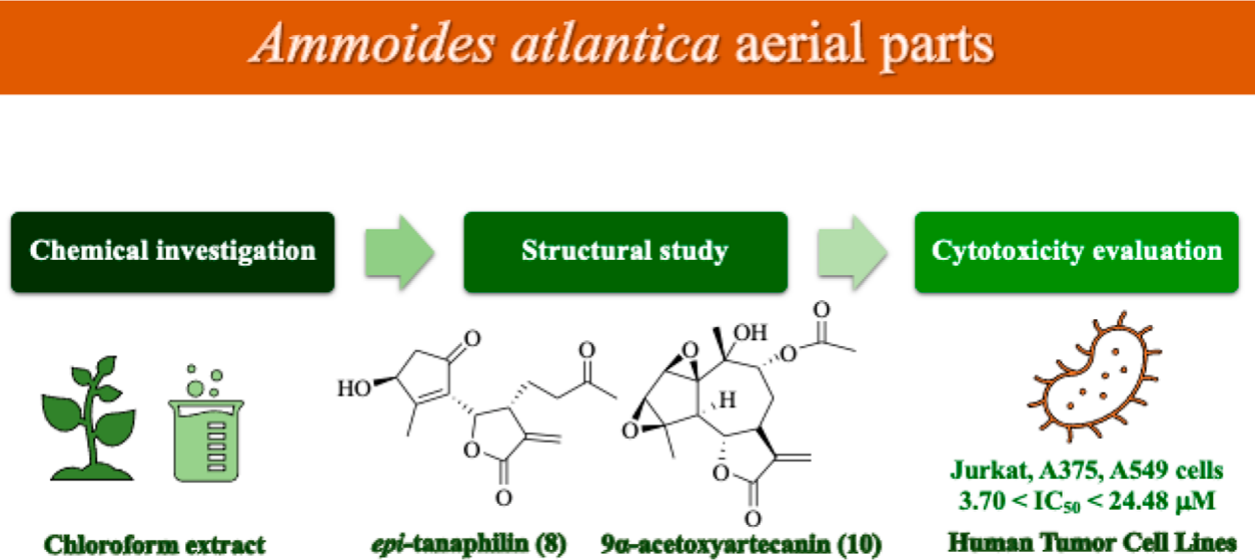

Seven new terpenoids, namely, guaiane (**1**–**4**), eudesmane (**5**), and bisabolane (**6**) sesquiterpenoids and a furanone (**7**), were isolated from the aerial parts of *Ammoides atlantica*, a herbaceous plant growing in Algeria, together with eight known compounds. All metabolites were characterized by their 1D and 2D NMR and HRESIMS data. A combined DFT/NMR method was applied to study the relative configurations of **1**–**4**, **6**, and **7**. All compounds, except **2**, were assayed against MCF-7, A375, A549, HaCaT, and Jurkat cell lines. Compounds **8**, **10**, and **11** induced a dose-dependent reduction in cell viability with different potency on almost all cell lines used. The most active compounds, **8** and **10**, were studied to assess their potential apoptotic effects and cell cycle inhibition.

In Algeria, the plant family Apiaceae consists of about 55 genera and 130 species. Among them, the genus *Ammoides* is represented only by two species, of which one, *A. atlantica* (Coss & Durieu) H. Wolff, is an endemic plant^1^ traditionally used as an infusion to treat headache, fever, diarrhea, and vitiligo^2^ and is also added as a spice in some recipes.

Flavonoids and terpenoids are indicated in the literature as typical components of the genus *Ammoides*.^3,4^ However, previous chemical investigations of the plant were mainly focused on the analysis of the essential oil composition^5−7^ and on the polar extract antioxidant^8^ and anti-inflammatory^9^ activities and phytochemical characterization,^10^ while no studies have been reported to date on the separation and chemical identification of the nonpolar constituents.

In the course of continuing studies on Algerian species^11,12^ aimed at the isolation of cytotoxic and/or antiangiogenic specialized metabolites, a phytochemical study of the aerial parts of the *A. atlantica* chloroform extract, guided by an analytical approach based on UHPLC-HRESI-Orbitrap/MS, was performed, leading to the isolation and structural characterization of seven new terpenoids, namely, four guaiane (**1**–**4**), an eudesmane (**5**), and a bisabolane (**6**) sesquiterpenoid and a furanone (**7**), together with eight known compounds belonging to the sesquiterpene and flavonoid classes. The relative stereostructures of some of these secondary metabolites, namely, **1**–**4**, **6**, and **7**, were assessed through a previously developed and optimized combined computational protocol (DFT/NMR),^13,14^ based on a comparison of the experimental ^13^C/^1^H NMR chemical shift data and the respective predicted values. A quantitative analysis of the main constituents of the cytotoxic chloroform extract was also carried out by means of LC-ESI/Orbitrap/MS.

Finally, all compounds were assayed against MCF-7 (human breast cancer), A375 (human malignant melanoma), A549 (human alveolar adenocarcinoma), Jurkat (human T-lymphocyte), and HaCaT (human epidermal keratinocyte) cell lines. The effect on apoptosis and cell cycle was also investigated for the two most active compounds found (**8** and **10**).

## Results and Discussion

**Chart 1:**
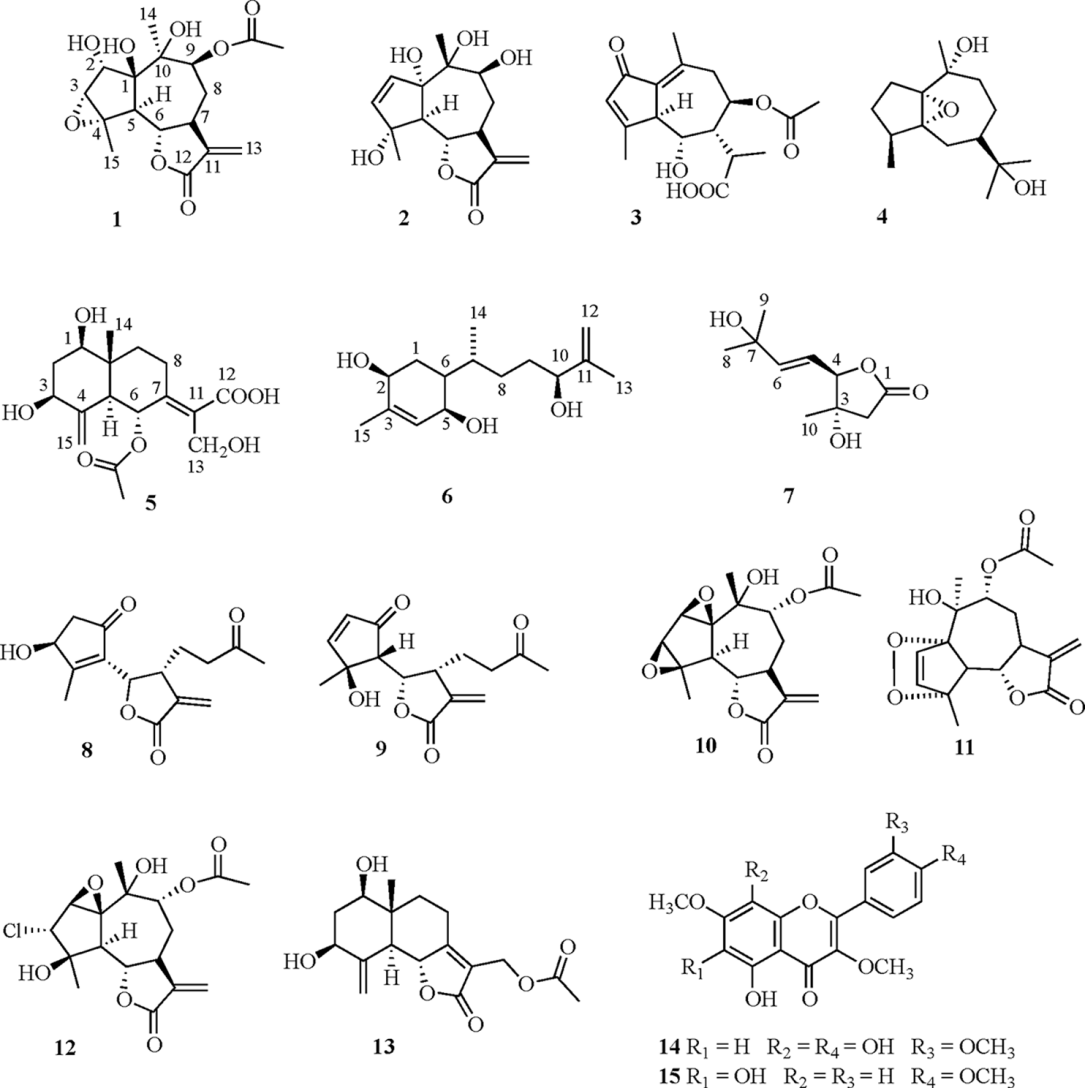


The aerial parts of *A. atlantica* were extracted with solvents of increasing polarity. The chloroform extract was investigated using a UHPLC-HRESI-Orbitrap/MS approach, indicating the presence of a series of sesquiterpenoids and polymethylated flavonoids previously unreported for this species. Thus, the extract was subjected to flash chromatography followed by RP-HPLC, to yield seven new (**1**–**7**) and eight known compounds (**8**–**15**) (Chart 1).

The molecular formula, C_17_H_22_O_8_, of compound **1** was determined from the sodiated molecular ion at *m*/*z* 377.1204 [M + Na]^+^ in its HRESIMS, requiring seven indices of hydrogen deficiency. The ^1^H NMR spectrum (Table 1) showed the presence of three tertiary methyl groups at δ_H_ 1.36, 1.38, and 2.14, of which one was attributed to an acetyl methyl. The ^13^C NMR spectrum (Table 1) exhibited signals attributable to two methyls, one methylene, an olefinic methylene, two methines, four hydroxymethines, an olefinic quaternary carbon, three oxygenated tertiary carbons, an acetyl group, and a lactone moiety. 1D TOCSY and COSY experiments established the spin systems H-2–H-3, H-5–H_2_-9, and H_2_-9–H_2_-13 included in an α-methylene-γ-lactone typical of guaianolides^15^ and suggested the presence of two oxygenated methines at C-2 and C-9. The presence of an epoxy ring was suggested by the methine signal, at δ_C_ 61.6 and δ_H_ 3.62 (1H, d, *J* = 3.0 Hz), and the resonance at δ_C_ 72.0 in the ^13^C NMR spectrum. An HSQC experiment was used to correlate all protons to the respective carbons, thereby confirming all the above assignments. The HMBC spectrum showed correlations between the methyl signal at δ 1.36 and C-2 and C-4, the methyl signal at δ_H_ 1.38 and C-1 and C-9, the methine at δ_H_ 2.39 and C-4, C-7, and C-10, the hydroxymethine at δ_H_ 5.11 and C-7 and C-10, the methylene at δ_H_ 5.60 and 6.16 and C-7, C-11, and C-12, the hydroxymethine at δ_H_ 4.61 and C-1, C-5, and C-8, and the hydroxymethine at δ_H_ 3.62 and C-1 and C-5 and aided in the location of the hydroxy groups at C-1, C-2, C-9, and C-10 and the epoxy ring at C-3 and C-4. An acetyl group at C-9 was inferred from the HMBC correlation between δ_H_ 5.11 (H-9) and δ_C_ 169.9 (CH_3_CO). The relative configuration of compound **1** was studied by experimental NOE analysis and a DFT/NMR computational method. Diagnostic NOE correlations were observed between H-2 and H-3, H-2 and Me-14, H-5 and Me-14, and H-9 and Me-14, showing that these protons were cofacial. To support the correct stereostructure, a density functional theory (DFT)/NMR computational procedure was applied,^13,14^ following four principal phases: (1) conformational sampling performed at the empirical theory level, through molecular dynamics (MD) and/or by Monte Carlo multiple minimum methods (MCMM) for each diastereoisomer under examination; (2) optimization of the geometry and energy at the quantum mechanical (QM) level; (3) single-point GIAO calculations at the QM level of ^13^C/^1^H NMR chemical shift parameters of all the structures; and (4) comparison of the Boltzmann-averaged NMR properties calculated for each stereoisomer with those experimentally measured for the compound under examination using the mean absolute error (MAE) as a statistical parameter to indicate the most probable stereoisomer. MCMM and MD simulations were performed to account for an extensive conformational search at the empirical level for each of the 16 possible diastereoisomers of **1**, using the OPLS force field (MacroModel, Schrödinger Suite 2021).^16^ In steps 2 and 3, the single-point GIAO calculations of ^13^C and ^1^H chemical shifts were performed on the nonredundant conformers using a MPW1PW91 functional and the 6-31G(d,p) basis set with IEFPCM for simulating the methanol solvent,^17,18^ for which the geometries were optimized previously at the same functional and 6-31G(d) basis set.^19^ Afterward, a comparison between the calculated and experimental ^13^C and ^1^H NMR chemical shifts for each diastereoisomer was evaluated by the Δδ parameter (Δδ = |δ_calc_ – δ_exp_|, the difference between experimental and calculated ^13^C and ^1^H NMR chemical shifts) and the MAE parameter [MAE = ∑[|δ_exp_–δ_calc_|]/*n*, the summation (∑) of the *n* computed absolute δ error values (Δδ), normalized to the number of Δδ errors considered (*n*)]. Compound **1p** showed the lowest ^13^C and ^1^H MAE values (3.0 and 0.11 ppm, respectively), indicating 1S*,2S*,3R*,4S*,9S*,10S* as the relative configuration for **1**. The analysis of the MAE value was combined with the experimental NOE data reported above. To confirm the findings obtained, the DP4+ method,^20,21^ a powerful tool for assigning the correct stereochemical patterns of organic compounds, was also employed, and the isomer **1p** showed the highest DP4+ probabilities (100.00%). Thus, the structure established for **1** was 1S*,2S*,10S*-trihydroxy-3R*,4S*-epoxy-9S*-acetoxy-5α,7αH-guaia-11(13)-en-12,6α-olide.

**Table 1:** ^1^H and ^13^C NMR Spectroscopic Data of Compounds **1**–**3**^a^

	**1**		**2**		**3**	
position	δ_H_	δ_C_	δ_H_	δ_C_	δ_H_	δ_C_
1		86.7		93.5		134.8
2	3.69 br s	78.1	5.85 d (6.0)	140.0		195.2
3	3.62 d (3.0)	61.6	5.90 d (6.0)	134.3	6.20 s	135.0
4		72.0		82.7		173.0
5	2.39 d (11.0)	56.6	2.72 d (11.0)	67.2	3.69 d (10.4)	52.3
6	4.61 dd (11.0, 10.0)	79.6	4.57 dd (11.0, 10.0)	83.0	3.86 br t (10.5)	79.2
7	3.15 m	42.9	3.39 m	39.1	2.56 br dd (11.5, 10.4)	59.0
8a	2.43 m	30.3	2.34 m	34.5	4.87^b^	71.7
8b	1.85 m		1.93 m			
9a	5.11 dd (5.0, 2.0)	80.0	3.99 br t (3.0)	80.6	2.89 dd (13.2, 11.0)	44.6
9b					2.43^b^	
10		71.9		77.0		147.5
11		139.0		140.8	2.68 m	41.1
12		170.1		172.1		179.0
13a	6.16 d (2.0)	119.0	6.22 d (2.5)	120.3	1.39 d (6.5)	15.9
13b	5.60 d (2.0)		5.68 d (2.5)			
14	1.38 s	22.7	1.02 s	22.8	2.44 s	20.9
15	1.36 s	22.9	1.42 s	22.0	2.36 s	20.9
CH_3_CO	2.14 s	20.0			2.13 s	20.0
CH_3_CO		169.9				171.0

a Spectra were recorded in methanol-*d*_4_ at 600 MHz; *J* values are in parentheses and reported in Hz; chemical shifts are given in ppm; assignments were confirmed by COSY, 1D-TOCSY, HSQC, and HMBC experiments.

b Overlapped signal.

Compound **2** (C_15_H_20_O_6_) displayed a sodiated molecular ion at *m*/*z* 319.1155 [M + Na]^+^, requiring six hydrogen deficiencies. Its NMR features suggested the presence of a guaianolide sesquiterpene.^15,22^ The NMR spectra (Table 2) showed the presence of two methyls, two methylenes (one olefinic), four methines (two olefinic), two hydroxymethines, three oxygenated tertiary carbons, one quaternary carbon, and a lactone group. 1D TOCSY, COSY, and HSQC experiments were useful to establish the spin systems, H-5–H-9 and H-6–H_2_-13, included in an α-methylene-γ-lactone unit. The HMBC spectrum showed correlations between the methyl signal at δ_H_ 1.02 and C-1 and C-9, the methyl signal at δ_H_ 1.42 and C-2, C-4, and C-5, the methine at δ_H_ 2.72 and C-1, C-4, C-6, and C-10, the hydroxymethine at δ_H_ 4.57 and C-8, and the olefinic methines at δ_H_ 5.85 and 5.90 and C-1, C-4, and C-5, hence locating the double bond at C-2,C-3 and the hydroxy groups at C-1, C-4, and C-9. Following the same computational protocol described above, also, in this case, the DFT/NMR protocol^13,14^ was used to suggest the relative configuration of this secondary metabolite; thus, **2i** showed the lowest ^13^C and ^1^H MAE values (2.10 and 0.12 ppm, respectively), indicating 1S*,4R*,9R*,10R* as the relative configuration for **2**. To confirm these findings, the DP4+ method, where the isomer **2i** showed the highest DP4+ probabilities (100.00%), was also employed. Thus, the structure established for **2** was 1S*,4R*,9R*,10R*-tetrahydroxy-5α,7αH-guaia-2(3),11(13)-dien-12,6α-olide.

**Table 2:** ^1^H and ^13^C NMR Spectroscopic Data of Compounds **4**–**6**^a^

	**4**		**5**		**6**	
position	δ_H_	δ_C_	δ_H_	δ_C_	δ_H_	δ_C_
1a		80.0	3.45 dd (12.0, 4.0)	76.0	1.72 m	31.1
1b					1.33 m	
2a	2.01^b^	29.0	2.15 m	40.7	3.96 m	67.0
2b	1.54^b^		1.57 dd (11.0, 2.0)			
3a	1.55^b^	28.7	4.00 dd (13.0, 6.0)	70.1		135.9
3b	1.23 m					
4	2.02^b^	36.0		146.1	5.52 br s	131.6
5		72.3	1.74 d (11.0)	53.7	3.89 d (9.5)	71.0
6a	2.44 d (14.0)	27.2	5.22 d (11.0)	79.6	1.75 m	42.1
6b	1.38 m					
7	1.69^b^	47.5		167.2	2.01 m	33.1
8a	1.70 m	30.2	3.08 br dd (14.0, 3.5)	23.9	1.41 m	33.6
8b	1.34 m		2.54 ddd (18.0, 14.0, 6.0)		1.24 m	
9a	2.01^b^	36.0	2.25 br dd (14.0, 5.0)	37.6	1.62 m	30.6
9b	1.53^b^		1.32 m			
10		75.2		40.0	4.03 br t (6.4)	78.3
11		74.7		121.1		147.6
12a	1.18 s	2657		174.2	4.94 br s	111.7
12b					4.83 br s	
13	1.14 s	25.3	4.31 s	53.5	1.74 s	18.3
14	1.28 s	24.5	0.93 s	10.6	0.85 d (6.5)	15.3
15a	1.05 d (6.2)	19.0	5.41 br s	106.2	1.81 s	20.9
15b			5.12 br s			
CH_3_CO			1.91 br s	23.0		
CH_3_CO				178.0		

a Spectra were recorded in methanol-*d*_4_ at 600 MHz; *J* values are in parentheses and reported in Hz; chemical shifts are given in ppm; assignments were confirmed by COSY, 1D-TOCSY, HSQC, and HMBC experiments.

b Overlapped signal.

The HRESIMS of compound **3** (*m*/*z* 321.1332 [M – H]^−^) and the ^13^C NMR data were consistent with a molecular formula of C_17_H_22_O_6_. The ^1^H NMR spectrum (Table 1) showed signals for a methyl doublet at δ_H_ 1.39 (*J* = 6.5 Hz), two methyl singlets linked to double bonds at δ_H_ 2.36 and 2.44, a hydroxymethine broad triplet at δ_H_ 3.86 (*J* = 10.5 Hz), a singlet for an olefinic proton at δ_H_ 6.20, and an acetyl group at δ_H_ 2.13. The ^13^C NMR spectrum (Table 1) displayed signals typical of a guaiane-type sesquiterpene acid with an α,β-unsaturated carbonyl group at δ_C_ 135.7, 172.9, and 198.2, a double bond at δ_C_ 134.8 and 147.5, a carboxylic acid unit at δ_C_ 179.8, two oxygen-bearing carbon resonances at δ_C_ 71.7 and 82.2, and an acetyl group at δ_C_ 171.0 and 20.0. The α,β-unsaturated carbonyl was proposed at the C-2/C-4 positions by the HMBC correlation peaks between H-3–C-2, H-3–C-5, H-5–C-3, H-5–C-4, and H-5–C-6. The C-1,C-10 positions of the double bond were deduced from the HMBC correlations of Me-14–C-1, Me-14–C-9, and Me-14–C-10, while the HMBC correlations between H_2_-9–C-8 and H-7–C-8 were used to locate the acetoxy group at C-8. Finally, the HMBC correlations between H-5–C-6 and Me-15–C-6 led to the location of the hydroxy group at C-6. Considering this multistep analysis, a tentative stereoassignment was proposed for **3**. Thus, the stereoisomer **3b** among the eight possible diastereoisomers endowed with 7R*, 8R*,11S* configuration patterns with ^13^C NMR and ^1^H MAE values (3.48 and 0.16 ppm, respectively) was suggested. Also in this case, a NOESY correlation between H-6 and H-7 was used to support the hypothesis proposed. Also in this case, the DP4+ method was used to corroborate the configurational assignment mode, where the isomer **3b** showed the highest DP4+ probabilities (99.880%). Therefore, compound **3** was elucidated as 2-oxo-6α-hydroxy-8R*-acetoxyguaia-1(10),3(4)-dien-12-oic acid.

Compound **4** gave a molecular formula of C_15_H_26_O_3_, according to the [M + Na]^+^ ion at *m*/*z* 277.1756 (calcd for 277.1774) in its HRESIMS, requiring three indices of hydrogen deficiency. Its ^1^H NMR spectrum (Table 2) showed the signals of three tertiary methyls (δ_H_ 1.14, 1.18, and 1.28) and one secondary methyl group (δ_H_ 1.05, d, *J* = 6.2 Hz). The ^13^C NMR experiment (Table 2) exhibited 15 carbon signals, attributable to four methyls, five methylenes, two methines, and four oxygenated tertiary carbons. A comparison between these carbon chemical shifts and those of compounds **1**–**3** and related guaianolides led to the conclusion that compound **4** possesses a guaianolide skeleton.^23^ A COSY experiment of **4** showed connectivities between H-2–Me-15 in ring A and between H-6–H-9 in ring B. The presence of an epoxy ring was supported by the signals at δ_C_ 81.0 (C-1) and 72.3 (C-5) in the ^13^C NMR spectrum. Moreover, two nonprotonated carbinol carbons (δ_C_ 75.0 and 74.7) were also observed in the ^13^C NMR spectrum.^23^ HMBC cross-peaks of Me-15 to C-3, C-4, and C-5, of H-6 to C-1, C-8, and C-11, of Me-12 and Me-13 to C-7 and C-11, and of Me-14 to C-1, C-9, and C-10 suggested that two hydroxy groups are linked to C-10 and C-11. The relative configuration of **4a** (1R*,4R*,5R*,7R*,10R*) was suggested as a tentative stereoassigment of **4**, on considering the ^13^C and ^1^H MAE values (2.44 and 0.19 ppm, respectively). Thus, **4** was characterized as 1R*,5R*-epoxy-guaian-10R*,11-diol.

Compound **5** was assigned a molecular formula of C_17_H_24_O_7_ by means of the HRESIMS (*m*/*z* 339.1440 [M – H]^−^). The ^1^H NMR spectrum (Table 2) displayed resonances for one methyl singlet (δ_H_ 0.93), one hydroxymethylene (δ_H_ 4.31), three hydroxymethines (δ_H_ 3.45, 4.00, and 5.22), one exocyclic methylene (δ_H_ 5.12 and 5.41), and an acetyl group (δ_H_ 1.91). The ^13^C NMR spectrum (Table 2) indicated that **5** contains a methyl, four methylenes (one olefinic), a hydroxymethylene, a methine, three hydroxymethines, four quaternary carbons, an acetyl, and a carboxylic group. All these above-mentioned signals suggested a eudesmane framework for **5**.^24^ Results obtained from 1D TOCSY and COSY experiments established the correlations of all protons showing the sequences H-1–H-3, H-5–H-6, and H-8–H-9. An HMBC experiment was helpful in defining the substituent locations; thus the exocyclic double bond was located at C-4,C-15 from the H_2_-15–C-3 and H_2_-15–C-5 correlations, the double bond was placed at C-7,C-11 through the H_2_-13–C-7 and H_2_-13–C-12 correlations, and the hydroxymethines were positioned at C-1, C-3, and C-6 as a result of the H-2–C-1, H-2–C-3, and H-5–C-6 correlations, respectively. The acetyl moiety was placed at C-6 as evidenced by the chemical shift of the H-6 signal (δ_H_ 5.22). The relative stereochemistry of compound **5** was proposed from the ^1^H NMR coupling constant values of H-1, H-3, H-5, and H-6 and compared with those reported for closely related eudesmanes in the literature.^24^ Consequently, compound **5** was proposed as 1β,3β,13-trihydroxy-6α-acetoxy-eudesma-4(15),7(11)-dien-12-oic acid.

The HRESIMS of **6** (molecular formula C_15_H_26_O_3_) gave a [M + Na]^+^ peak at *m*/*z* 277.1774. The ^13^C NMR spectrum (Table 2) confirmed the presence of 15 carbons that were sorted as three methyls, four methylenes (one olefinic), six methines (including three oxygenated and one olefinic), and two quaternary carbons. The ^1^H NMR spectrum showed signals for two methyl group singlets at δ_H_ 1.74 and 1.81, one methyl doublet at δ_H_ 0.85 (*J* = 6.5 Hz), one exocyclic methylene at δ_H_ 4.83 and 4.94, one sp^2^ proton broad singlet at δ_H_ 5.52, three oxygenated methines at δ_H_ 3.89, 3.96, and 4.03, and signals for methylenes and methines in the region between δ_H_ 1.20 and 2.10. Results obtained from the COSY spectrum established the proton correlations of compound **6**, permitting the establishment of the spin systems H-1–H-2 and H-4–H-10, leading to the proposal of the presence of a bisabolene sesquiterpene.^15,25^ The HSQC and HMBC spectra also assisted in assigning most of the substituents: in particular, the methyl signal at δ_H_ 1.74, showing an HMBC correlation with the carbon signal at δ_C_ 78.3 (C-10), was used to locate a hydroxy group at C-10. The hydroxy group at C-2 was indicated by the HMBC correlation between H-4–C-2 and Me-15–C-2, while the hydroxy group at C-5 was deduced by the HMBC correlations between H-4–C-5. Following the same procedures reported above, diastereoisomer **6d** (2S*,5R*,6R*,7S*,10S*) showed a better fit with the experimental data (2.14 and 0.11 ppm as ^13^C and ^1^H MAE values, respectively, and 100.00% as DP4+ probability value). Thus, compound **6** was assigned the proposed structure of 2S*,5R*,10S*-trihydroxybisabol-3,12-diene.

Compound **7** was assigned the molecular formula C_10_H_16_O_4_ (*m*/*z* 223.0942 [M + Na]^+^) by HRESIMS. Analysis of its 1D and 2D NMR spectra (see Experimental Section) revealed **7** to have three methyl singlets (δ_H_ 1.31 and 1.32), a methylene (δ_H_ 2.53, 2.69, d, *J* = 16.5 Hz), three methines (two olefinic) (δ_H_ 4.74, d, *J* = 8.0 Hz, 5.67, dd, *J* = 16.0, 8.0 Hz and 5.97, d, *J* = 16.0 Hz), two oxygenated tertiary carbons, and a lactone. All proton and carbon signals were accurately assigned by means of HSQC and HMBC experiments. In particular, the HMBC correlations between the methyl signal at δ_H_ 1.31 and C-2, C-3, and C-4 allowed the location of an oxygenated tertiary carbon at C-3, while the HMBC cross-peaks between the signal at δ_H_ 5.67 and C-4 and C-7 indicated the occurrence of a five-membered side chain linked at C-4 with a terminal oxygenated tertiary carbon. Finally, the HMBC correlations between δ_H_ 4.74 and C-1, C-3, and C-6 and δ_H_ 2.53 and 2.69 and C-1 supported the lactone being in a 2(3*H*)-furanone ring. Compound **7a** showed the lowest ^13^C and ^1^H MAE values (1.17 and 0.08 ppm, respectively), indicating 3S*,4R* as the relative configuration for **7**. To confirm these findings, the DP4+ method was also employed, where the isomer **7a** showed the highest DP4+ probabilities (100.00%). In light of these data, the structure of **7** was elucidated as dihydro-3S*-hydroxy-3S*-methyl-4R*-(3-hydroxy-3-methyl-1-buten-1-yl)-2(3*H*)-furanone.

The remaining isolated compounds were characterized as the sesquiterpenes *epi*-tanaphilin (**8**),^26^*seco*-tanapartholide B (**9**),^27^ 9α-acetoxyartecanin (**10**),^15^ apressin (**11**),^28^ 3α-chloro-9α-acetoxy-4β,10α-dihydroxy-1β,2β-epoxy-5α,7αH-guai-11(13)-en-12,6α-olide (**12**),^15^ and 1β,3β-dihydroxy-13-acetoxy-eudesma-4(15),7(11)-dien-12,6α-olide (**13**)^24^ and the polymethylated flavonoids gossypetin 3,7,3′-trimethyl ether (**14**)^29^ and tanetin (**15**),^30^ by NMR and MS analysis and comparison of their data with those reported in the literature. Furthermore, the relative stereoassigment of *seco*-tanapartholide B (**9**) as 4S*,5R*,6R*,7R* (2.12 and 0.17 for ^13^C and ^1^H MAE values, respectively) was suggested.

**Figure 1:**
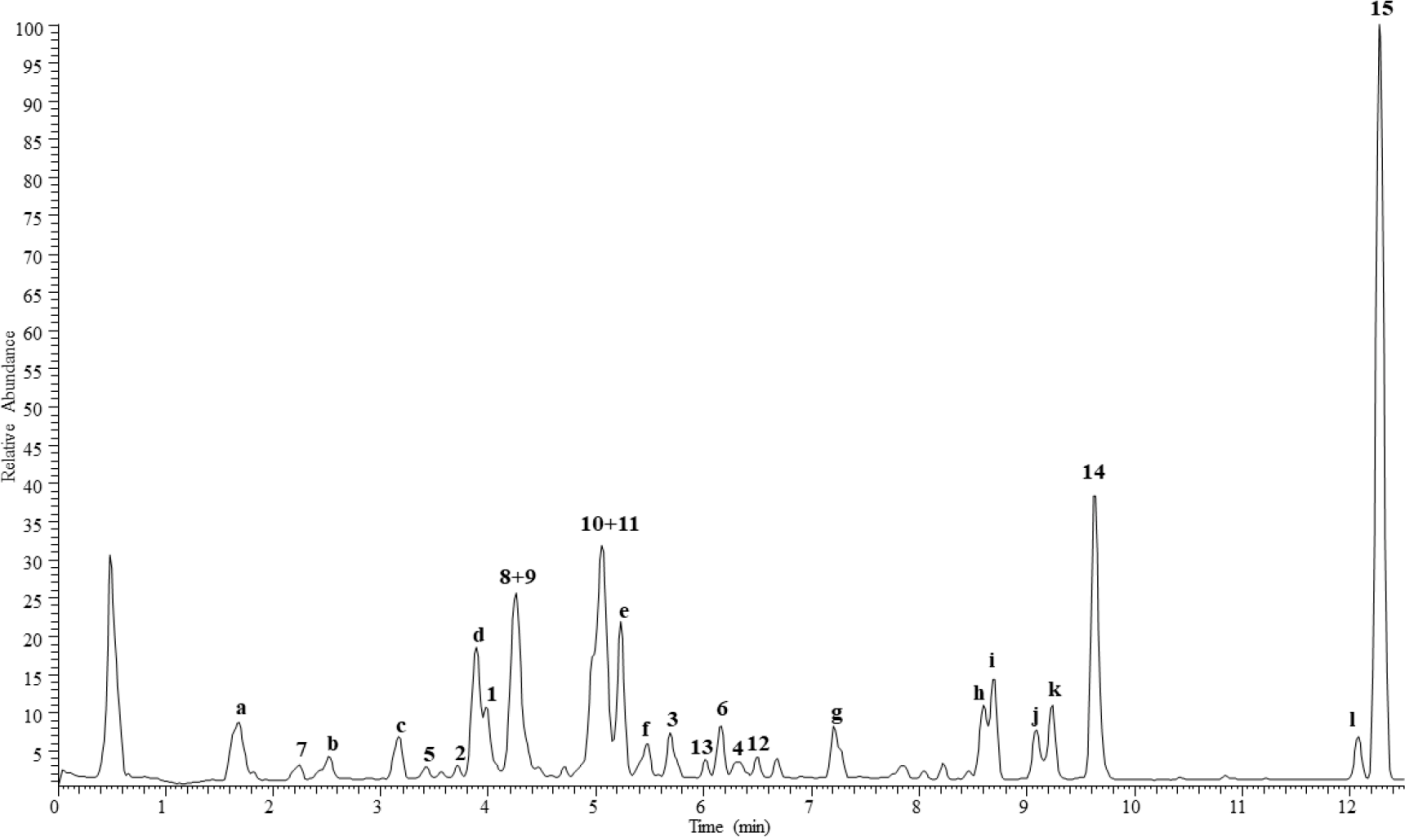
UHPLC-HRESIMS profile of the chloroform extract of *A. atlantica* aerial parts. Peak numbers correspond to those of Chart 1. **a** = carboxylic acid; **b** = unidentified; **c** = isomer of **1**; **d** = dihydroxy-dodecadienoic acid; **e** = hydroxy-decatrienoic acid; **f** = isomer of **10** and **11**; **g** = sesquiterpene; **h**, **j** = sesquiterpene isomers; **k**, **l** = methoxylated flavonoids; **j** = sesquiterpene dimer.

The chemical profile of the chloroform extract from *A. atlantica* aerial parts was investigated by UHPLC-HRESI-Orbitrap/MS. In agreement with results obtained through the isolation process, the major components were represented by terpenoids (peaks **1**–**13**). In addition, the two methoxylated flavonoid aglycones (peaks **14** and **15**) were also detected in the last region of the chromatogram (Figure 1). All compounds were identified based on full MS and MS/MS data (Table S1, Supporting Information) and injection of isolates as reference standards. Several minor peaks (**a**–**l**) were identified tentatively since the molecules hypothesized were not isolated from the extract, but only detected by analytical investigation. Peak **a** showed a deprotonated molecular ion [M – H]^−^ at *m*/*z* 155.0345, for which the fragmentation generated an intense base ion peak at *m*/*z* 111.04, due to the loss of a carboxylic unit, suggesting **a** to be an organic acid. Peak **c** showed the same full HRESIMS profile of **1** with a deprotonated molecular ion at [M – H]^−^ at *m*/*z* 353.1243 and two adduct ions [M + Cl]^−^ and [M + HCOOH]^−^ at *m*/*z* 389.1011 and 399.1297, respectively, suggesting **c** as an isomer of **1**. Full MS ([M – H]^−^ at *m*/*z* 227.1287) and MS/MS of peak **d** ([M – H – H_2_O]^−^ and ([M – H – 2H_2_O]^−^ at *m*/*z* 209.12 and 191.11, respectively) were in agreement with the structure of a dihydroxy-dodecadienoic acid. Peak **e** was identified as a hydroxy-decatrienoic acid, as deduced by the [M – H]^−^ at *m*/*z* 181.0866 and fragment ions at *m*/*z* 137.10 ([M – H – CO_2_]^−^) and 119.09 ([M – H – CO_2_ – H_2_O]^−^). Peak **f** could be proposed as an isomer of **10** and **11** based on the high similarity between their full and MS/MS spectra. The full MS of peak **g** showed adduct ions [M + Cl]^−^ and [M + HCOOH]^−^ at *m*/*z* 303.1369 and 313.1659, respectively, and a deprotonated molecular ion [M – H]^−^ at *m*/*z* 267.1603 that generated a fragment ion at *m*/*z* 249.15 due to the loss of a water molecule, suggesting the occurrence of a sesquiterpene with at least one hydroxy group. Similarly, peaks **h** and **i** showed the same adduct ions [M + Cl]^−^ and [M + HCOOH]^−^ at *m*/*z* 423.1663 and 413.1374, respectively, and a deprotonated molecular ion [M – H]^−^ at *m*/*z* 377.1607, while the fragmentation MS displayed some product ions in common with compounds **10** and **11** (*m*/*z* 231.10, 213.09, 195.08, 171.08, 143.05, 123.04, and 93.03), suggesting the occurrence of two further sesquiterpene isomers having two acetyl groups ([M – H – 60 – 60]^−^ at *m*/*z* 257.08). MS/MS experiments on peak **k** (deprotonated molecular ion [M – H]^−^ at *m*/*z* 329.0667) generated the loss of two methyl groups (product ions at *m*/*z* 314.04 and 299.02); thus it was annotated as a dimethylated flavonoid. Similarly, peak **l** showed a deprotonated molecular ion [M – H]^−^ at *m*/*z* 313.0719 and two product ions at *m*/*z* 298.05 and 283.02, indicating the presence of two methyl groups on a flavonoid skeleton. Finally, peak **j** showed a deprotonated molecular ion [M – H]^−^ at *m*/*z* 523.2339 and a complex fragmentation pathway leading to being proposed as a sesquiterpene dimer (hypothesized molecular formula C_30_H_36_O_8_).

**Figure 2:**
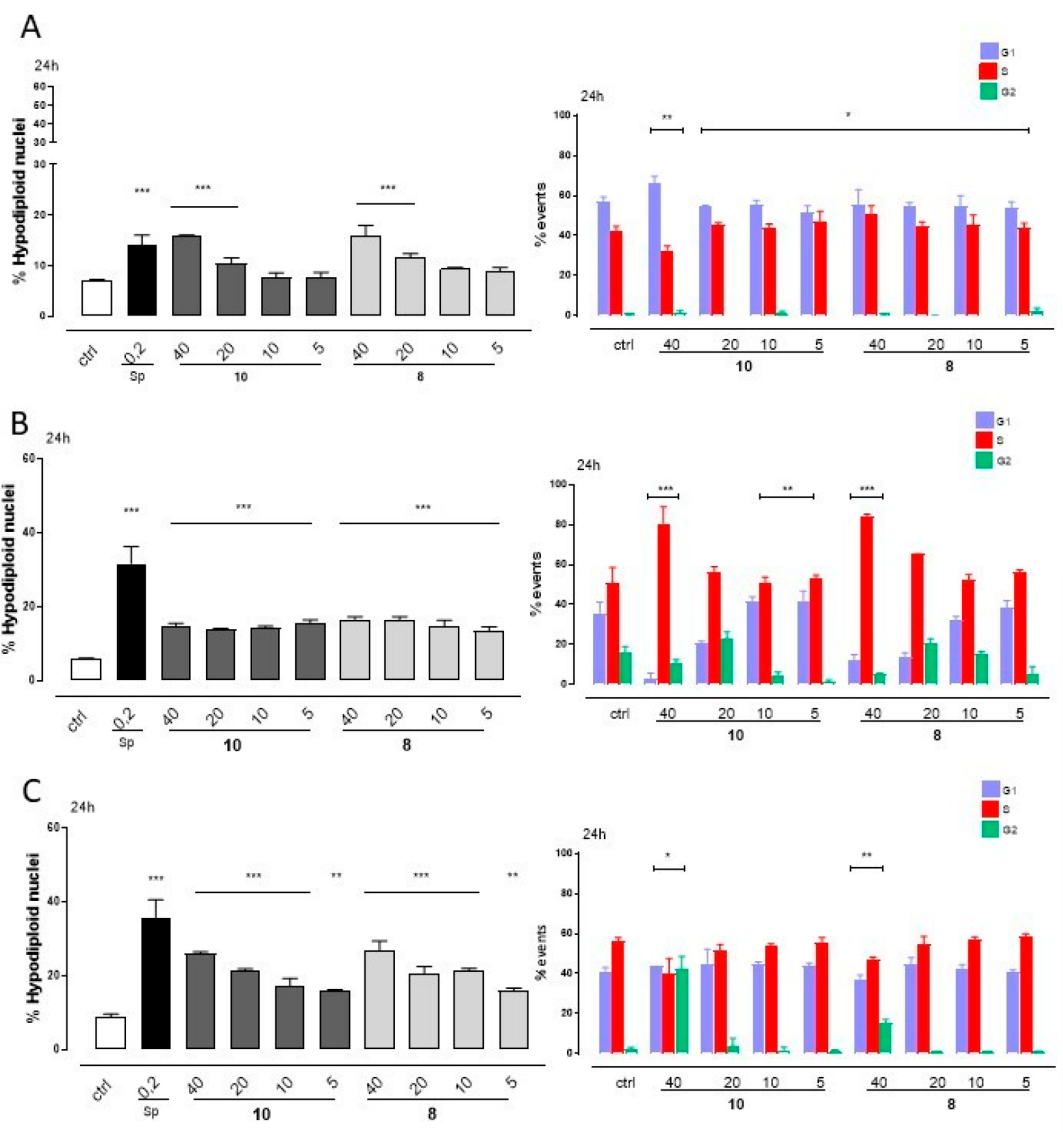
Hypodiploid nuclei and cell cycle analysis of DNA content, with propidium iodide staining, were evaluated by a flow cytometric assay on A549 (panel A), Jurkat (panel B), and HaCaT (panel C) cells treated, respectively, with compound **10** or **8** (both 40–20–10–5 μM) for 24 h. Staurosporine (Sp) at 0.2 μM was used as a positive control. Results are expressed as means ± SEM of three independent experiments each performed in triplicate. Data were analyzed by the nonparametric Mann–Whitney U test. *p < 0.05, **p < 0.005, and ***p < 0.01 *vs* nontreated cells.

Despite the wide biological activity of sesquiterpenoids, the low specificity of the Michael-type addition reaction represents a limitation for the use of these classes of compounds as therapeutic agents, due to their toxicity. On the other hand, several sesquiterpenoids have demonstrated to interact specifically with different molecular targets and to possess properties for drug-like compounds. Thus, these molecules, despite the toxicity of several derivatives, could be good candidates for the development of antitumor, anti-inflammatory, and antimicrobial drugs.^31^ In light of the above considerations, four human tumor cell lines (MCF-7, A375, A549, and Jurkat) and nontumor HaCaT cells were used to evaluate the cytotoxic activity of compounds **1** and **3**–**13** (40, 20, 10, and 5 μM) using an MTT ([3-(4,5-dimethylthiazol-2-yl)-2,5-diphenyltetrazolium bromide]) assay. Compound **2** was not tested since it was isolated in too small an amount. The results indicated that compounds **8**, **10**, and **11** induced a significant dose-dependent reduction in cell viability on most of the cell lines, with A549, A375, and Jurkat being more susceptible to these tested compounds (Table 3). Based on the data obtained from the viability test, the potential apoptotic effect and the evaluation of the cell cycle distribution were investigated for the most active and abundant compounds **8** and **10**, with the A549 and Jurkat tumor cell lines and the nontumorigenic HaCaT cell line. With A549 and HaCaT cells, both compounds caused a significant increase in hypodiploid nuclei, after 24 h of treatment. In HaCaT cells these two compounds induced cells accumulating in the G2 phase at 40 μM, probably due to the high toxicity at this concentration. In Jurkat cells, a major proliferative capacity of these cells was confirmed by an increase of cell cycle S phase for both compounds in a dose-dependent manner. In agreement with these results, compound treatment with all three cell lines induced a significant (*p* < 0.001) increase of apoptotic response in a dose-dependent manner, as depicted by the hypodiploid nuclei in Figure 2.

**Table 3:** IC_50_ (μM) of Compounds **1** and **3**–**13** Using the MTT Assay^a^

compound	Jurkat	A549	A375	MCF-7	HaCaT
**1**	>10	>10	>10	>10	>10
**3**	>10	>10	>10	>10	>10
**4**	>10	>10	>10	>10	>10
**5**	>10	>10	>10	>10	>10
**6**	>10	>10	>10	>10	>10
**7**	>10	>10	>10	>10	>10
**8**	7.7 ± 0.45	8.9 ± 0.60	>10	>10	2.9 ± 0.57
**9**	>10	>10	>10	>10	>10
**10**	5.0 ± 0.59	>10	3.7 ± 0.47	9.6 ± 0.52	1.6 ± 0.21
**11**	4.7 ± 0.45	4.1 ± 0.11	>10	>10	1.7 ± 0.55
**12**	>10	>10	>10	>10	3.5 ± 0.27
**13**	>10	>10	>10	>10	>10
Sp^b^	3.2 ± 0.93	9.1 ± 1.20	2.1 ± 0.90	6.3 ± 0.55	1.6 ± 0.60

a Data are expressed as IC_50_ (μM) values indicating the concentration of each compound that inhibits cell growth by 50% as compared to control cells.

b Sp: Staurosporin (0.2 μM) was used as a positive control.

Considering the potential activity demonstrated by compounds **8**, **10**, and **11**, a quantitative LC-MS analysis of the main components isolated from the CHCl_3_ extract was performed, recording high-resolution MS/MS data in PRM mode, useful for the targeted substance quantification. The results obtained confirmed sesquiterpenes being the major plant specialized metabolites present (2.55 ± 0.21 g/100 g DW), followed by methylated flavonoids (0.62 ± 0.09 g/100 g DW, Table S2, Supporting Information). Furthermore, the most abundant constituents were represented by the sesquiterpenes **8**, **9**, **10**, and **11**, while tanetin (**15**) was the most abundant flavonoid.

The present investigation has provided detailed information about the chemical composition of the nonpolar extract of *A. atlantica* aerial parts, highlighting the presence of cytotoxic sesquiterpenoids and methylated flavonoids. These findings suggested that this plant could be considered as a potential source of bioactive compounds and could provide scientific data to obtain more safe traditional medicinal plant preparations.

## Experimental Section

### General Experimental Procedures

Optical rotations were measured on an Atago AP-300 digital polarimeter with a 1 dm microcell and a sodium lamp (589 nm). NMR data were recorded on a Bruker DRX-600 spectrometer (Bruker BioSpinGmBH, Rheinstetten, Germany) equipped with a Bruker 5 mm TCI Cryoprobe at 300 K. All 2D NMR spectra were acquired in methanol-*d*_4_, and standard pulse sequences and phase cycling were used for the TOCSY, COSY, NOESY, HSQC, and HMBC spectra obtained. Data were processed with Topspin 3.2 software. HRESIMS data were measured on a Q Exactive Plus mass spectrometer, using an Orbitrap-based FT-MS system, equipped with an ESI source (ThermoFisher Scientific Inc., Bremen, Germany). Column chromatography was performed over silica gel (70–220 mesh, Merck, Germany). RP-HPLC separations were carried out using a Shimadzu LC-8A series pumping system equipped with a Shimadzu RID-10A refractive index detector and a Shimadzu injector (Shimadzu Corporation, Japan) on a C_18_ μ-Bondapak column (30 × 7.8 mm, 10 μm, Waters, Milford, MA, USA) and a mobile phase consisting of a MeOH–H_2_O mixture at a flow rate of 2.0 mL/min. TLC separations were conducted using silica gel 60 F_254_ (0.20 mm thickness) plates (Merck, Germany) and Ce(SO_4_)_2_–H_2_SO_4_ as spray reagent (Sigma-Aldrich, Italy).

### Plant Material

The aerial parts of *A. atlantica* were collected in March 2016, in the Jijel Region, Algeria. The plant was identified by Dr. Jijar Dibilaire, and a voucher specimen (131AAT/VAREBIOL/451) was deposited in the Herbarium of the Chemistry Department, University of Constantine 1, Algeria.

### UHPLC-HRESI-Orbitrap/MS/MS Analysis

UHPLC-HRESIMS/MS was performed using a Vanquish Flex binary pump LC system coupled with a Q Exactive Plus MS, using a C_18_ Kinetex biphenyl column (100 × 2.1 mm, 2.6 μm, Phenomenex, Italy) provided with a Security Guard Ultra cartridge, eluting with formic acid in acetonitrile 0.1% v/v (solvent A) and formic acid in H_2_O 0.1% v/v (solvent B) and developing a solvent gradient from 5 to 55% A within 14 min, at a flow rate 0.5 mL/min. The column oven and autosampler temperatures were maintained at 35 and 4 °C, respectively. Full spectra (70 000 resolution, 220 ms maximum injection time) and data dependent-MS/MS (17 500 resolution, 60 ms maximum injection time) were acquired in the negative-ionization mode in a scan range of *m*/*z* 120–1200 using ionization parameters as previously reported.^32^

### Extraction and Isolation

The dried aerial parts of *A. atlantica* (120 g) were extracted with solvents of increasing polarity, including *n*-hexane, CHCl_3_, and MeOH, by exhaustive maceration (1 L), to give 1.5, 5.4, and 9 g of the respective dried residue. Part of the CHCl_3_ extract (4.6 g) was subjected to column chromatography (5 × 180 cm, collection volume 25 mL) over silica gel, eluting with *n*-hexane followed by increasing concentrations of CHCl_3_ in *n*-hexane (between 1% and 100%) continuing with CHCl_3_ followed by increasing concentrations of MeOH in CHCl_3_ (between 1% and 100%) and gathering 12 major fractions (A–L), together with pure compound **14** (7.0 mg). Fraction D (552.0 mg) was purified by RP HPLC with MeOH–H_2_O (47:53) as eluent to give compounds **11** (1.5 mg, *t*_R_ 12 min), **4** (1.5 mg, *t*_R_ 23 min), **3** (3.0 mg, *t*_R_ 24 min), and **15** (10.9 mg, *t*_R_ 52 min). Fraction E (147.0 mg) was submitted to RP-HPLC with MeOH–H_2_O (2:3) as eluent to yield compound **6** (1.2 mg, *t*_R_ 48 min). Fraction F (290 mg) was separated by RP-HPLC eluting with MeOH–H_2_O (35:65) to give **10** (3.9 mg, *t*_R_ 28 min) and **12** (1.9 mg, *t*_R_ 56 min). Fraction G (390 mg) was separated by RP-HPLC eluting with MeOH–H_2_O (3:7) to give compounds **1** (4.2 mg, *t*_R_ 10 min), **8** (3.8 mg, *t*_R_ 18.0 min), **9** (3.0 mg, *t*_R_ 19 min), and **10** (10.1 mg, *t*_R_ 35 min). Fractions I (144.5 mg), J (218.0 mg), K (160 mg), and L (207 mg) were separately subjected to RP-HPLC eluting with MeOH–H_2_O (1:4) to give **8** (2.0 mg, *t*_R_ 48 min) from fraction I, **1** (3.3 mg, *t*_R_ 29 min) and **2** (0.8 mg, *t*_R_ 40 min) from fraction J, **7** (1.7 mg, *t*_R_ 28 min), **1** (0.3 mg, *t*_R_ 30 min), and **8** (3.3 mg, *t*_R_ 44 min) from fraction K, and **5** (1.2 mg, *t*_R_ 13 min), **8** (1.7 mg, *t*_R_ 52 min), and **13** (1.7 mg, *t*_R_ 100 min) from fraction L, respectively.

#### Compound **1**:

amorphous powder; [α]^25^_D_ +29 (*c* 0.03, MeOH); ^1^H and ^13^C NMR, see Table 1; HRESIMS *m*/*z* 377.1204 [M + Na]^+^ (calcd for C_17_H_22_O_8_Na, 377.1212).

#### Compound **2**:

amorphous powder; [α]^25^_D_ −5 (*c* 0.1, MeOH); ^1^H and ^13^C NMR, see Table 1; HRESIMS *m*/*z* 319.1155 [M + Na]^+^ (calcd for C_15_H_20_O_6_Na, 319.1158).

#### Compound **3**:

amorphous powder; [α]^25^_D_ +48 (*c* 0.06, MeOH); ^1^H and ^13^C NMR, see Table 1; HRESIMS *m*/*z* 321.1332 [M – H]^−^ (calcd for C_17_H_21_O_6_, 321.1338).

#### Compound **4**:

amorphous powder; [α]^25^_D_ +4 (*c* 0.1, MeOH); ^1^H and ^13^C NMR, see Table 2; HRESIMS *m*/*z* 277.1756 [M + Na]^+^ (calcd for C_15_H_26_O_3_Na, 277.1774).

#### Compound **5**:

amorphous powder; [α]^25^_D_ +30 (*c* 0.1, MeOH); ^1^H and ^13^C NMR, see Table 1; HRESIMS *m*/*z* 339.1440 [M – H]^−^ (calcd for C_17_H_23_O_7_, 339.1444).

#### Compound **6**:

amorphous powder; [α]^25^_D_ −20 (*c* 0.1, MeOH); ^1^H and ^13^C NMR, see Table 3; HRESIMS *m*/*z* 277.1774 [M + Na]^+^ (calcd for C_15_H_26_O_3_Na, 277.1780).

#### Compound **7**:

amorphous powder; [α]^25^_D_ −35 (*c* 0.1, MeOH); ^1^H NMR (CD_3_OD, 600 MHz) δ_H_ 1.31 (3H, s, Me-10), 1.32 (6H, s, Me-8 and Me-9), 2.53 (1H, d, *J* = 16.5 Hz, H-2b), 2.69 (1H, d, *J* = 16.5 Hz, H-2a), 4.74 (1H, d, *J* = 8.0 Hz, H-4), 5.67 (1H, dd, *J* = 16.0, 8.0 Hz, H-5), 5.97 (1H, d, *J* = 16.0 Hz, H-6); ^13^C NMR (CD_3_OD, 600 MHz) δ_C_ 23.0 (C-10), 29.9 (C-8 and C-9), 43.0 (C-2), 70.7 (C-7), 77.0 (C-3), 90.8 (C-4), 121.7 (C-5), 143.3(C-6), 177.3 (C-1); HRESIMS *m*/*z* 223.0942 [M + Na]^+^ (calcd for C_10_H_16_O_4_Na, 223.0946).

### Computational Details and Determination of Relative Compound Configurations

Maestro and LigpPrep (Maestro, Schrödinger Suite 2021; LigpPrep, Schrödinger Suite 2021)^33,34^ were used for generating the starting 3D chemical structures of the possible relative diastereoisomers of compounds **1**–**4**, **6**, and **7** (Chart 1). As a first step, exhaustive conformational searches at the empirical MM level with the MCMM method (50 000 steps) and the LMCS method (50 000 steps) were performed in order to allow a full exploration of the conformational space.^13,14^ Furthermore, molecular dynamics simulations were performed at different temperatures (450, 600, 700, 750 K), with a time step of 2.0 fs, an equilibration time of 0.1 ns, and a simulation time of 10 ns. All the conformers obtained from the conformational searches were minimized using the OPLS (Optimized Potentials for Liquid Simulation) force field and the Polak–Ribier conjugate gradient algorithm. The “Redundant Conformer Elimination” module of Macromodel (MacroModel, Schrödinger Suite 2021)^16^ was used to select nonredundant conformers. All the above-mentioned QM calculations were performed using Gaussian 09 software.^18^ In detail, the obtained conformers were optimized at the QM level using the MPW1PW91 functional and the 6-31G(d) basis set^19^ in methanol (IEFPCM) to reproduce the effect of the experimental solvent. The selected conformers for the different diastereoisomers were accounted for in the subsequent computation of the ^13^C and ^1^H NMR chemical shifts, using the MPW1PW91 functional and the 6-31G(d,p) basis set. Final NMR parameter (chemical shift) values for each of the investigated diastereoisomers were built considering the influence of each conformer on the total Boltzmann distribution taking into account the relative energies. Final ^13^C and ^1^H NMR chemical shift sets of data for each of the diastereoisomers were extracted and computed considering the influence of each conformer on the total Boltzmann distribution considering the relative energies. Calibrations of calculated ^13^C and ^1^H NMR chemical shifts were performed following the multistandard approach (MSTD).^35,36^ Also, sp^2 13^C and ^1^H NMR chemical shifts were computed using benzene as a reference compound, while TMS was used for computing sp^3 13^C and ^1^H chemical shift data. Experimental and calculated ^13^C and ^1^H NMR chemical shifts were compared by computing the Δδ parameter: Δδ = |δ_exp_ – δ_calc_|, where δ_exp_ (ppm) and δ_calc_ (ppm) are the ^13^C/^1^H experimental and calculated chemical shifts, respectively. The MAEs for all the considered diastereoisomers were computed using the following equation:  defined as the summation (∑) of the *n* computed absolute error values (Δδ), normalized to the number of chemical shifts considered (*n*). Furthermore, DP4+ probabilities related to all the stereoisomers for each compound were computed by considering both ^1^H and ^13^C NMR chemical shifts and comparing them with the related experimental data.

### Quantitative Analysis

For the quantitative analysis of the main components, the most abundant isolated compounds were used for constructing calibration curves. Compounds **8**, **11**, and **14** were used as external standards for quantification of the *seco*-tanapartholides (**8** and **9**), the acetylated sesquiterpenoids (**1**, **3**, **10**–**12**), and the methoxylated flavonoids (**14** and **15**), respectively. Stock acetonitrile solutions (1 mg/mL) were prepared and successive solutions at different concentrations were obtained in triplicate by serial dilution. Calibration curves were constructed using concentration (range 0.50–0.015 mg/mL) with respect to the areas obtained by integration of MS peaks operating in the parallel reaction monitoring (PRM) mode (17 500 resolution, normalized collision energy 50%, maximum injection time 65 ms). Linear simple correlation was considered to analyze the relation between variables (*R*^2^ = 0.9946 for **8**; *R*^2^ = 0.9982 for **11**, and 0.9716 for **14**). Microsoft Office Excel was used to obtain the amount, finally expressed as g/100 g ± standard deviation (SD) of dry weight (DW).

### Cell Culture

Breast cancer (MCF-7), malignant melanoma (A375), alveolar adenocarcinoma (A549), and epidermal keratinocyte (HaCaT) human cell lines were cultured in Dulbecco’s modified Eagle’s medium containing 10% fetal bovine serum (FBS), supplemented with 100 U/mL each of penicillin and streptomycin and 2 mM l-glutamine and grown at 37 °C under a 5% CO_2_ air humidified atmosphere. The leukemia cell line (Jurkat) was maintained in RPMI medium supplemented with 10% FBS, 2 mM l-glutamine, 100 U/mL penicillin, and 100 mg/mL streptomycin at 37 °C in 5% CO_2_.

### Cell Viability

Cell viability was evaluated using a colorimetric assay based on an MTT assay, in order to compare the effect of potentially cytotoxic substances with a control. Briefly, cells were plated in 96-well tissue culture plates (3.5 × 10^3^ cells/well), and, after 24 h, the medium was replaced with a fresh one alone or containing serial dilutions of compounds **1** and **3**–**13** (40–20–10–5 μM), and the incubation was performed for 48 h. Staurosporine (0.2 μM) was used as the positive control. At the end of the treatment, 25 μL of MTT (5 mg/mL) was added to each well and the cells were incubated for an additional 3 h to allow the formation of a purple formazan precipitate; then, 100 μL of a solution containing 50% (v/v) *N*,*N*-dimethylformamide, and 20% (w/v) sodium decyl sulfate with an adjusted pH of 4.5 was added.^37^ The optical density (OD) of each well was measured with a microplate spectrophotometer (Multiskan Spectrum Thermo Electron Corporation reader) equipped with a 620 nm filter. Cell vitality was calculated as % vitality = 100 × (OD_treated_/OD_DMSO_).

### Apoptosis and Cell Cycle Analysis

The effect of compounds **8** and **10** on cell death was analyzed by propidium iodide (PI) (Sigma-Aldrich) staining and flow cytometry. Cells were plated at a density of 3 × 10^4^ cells/well in a 24-well plate. After 24 h, serial dilutions of compounds **8** and **10** (40–20–10–5 μM) were added and cells were recultured for 24 h. Staurosporine (0.2 μM) was used as a positive control. For apoptosis analysis cells were washed twice with phosphate-buffered saline (PBS) and incubated in 500 μL of a solution containing 0.1% Triton X-100, 0.1% sodium citrate, and 50 mg/mL PI, at 4 °C for 30 min in the dark. The PI-stained cells were subsequently analyzed by flow cytometry by FACS using CellQuest software. Data are expressed as the percentage of cells in the hypodiploid region. Cellular debris was excluded from the analysis by raising the forward scatter threshold, and the DNA content of the nuclei was registered on a logarithmic scale. Cell cycle profiles were evaluated by DNA staining with PI solution using a flow cytometer.^38^ Results from 10 000 events per sample were collected, and the relative percentage of the cells in G0/G1, S, and G2/M phases of the cell cycle was determined using the ModFit LT version 3.3 analysis software (BD Biosciences).

### Data Analysis

Data are reported as mean ± SEM values of independent experiments, performed at least three times, with three or more independent observations. Statistical analysis was performed by the nonparametric Mann–Whitney U test. Differences with *p* < 0.05 were considered statistically significant.

## Supporting Information Available

HRESIMS and NMR spectra of compounds **1**–**7**, UHPLC-HRESIMS data of the chloroform extract components, quantitative amounts of constituents isolated, and experimental and calculated NMR data of compounds **1**, **2**, **6**, and **7** (PDF)

## Dedication

Dedicated to Dr. William H. Gerwick, University of California at San Diego, for his pioneering work on bioactive natural products.
