# Supplementary material for: Cytotoxic Sesquiterpenoids from Ammoides atlantica Aerial Parts
Source: J Nat Prod. 2022 Feb 23;85(3):647–56. doi: 10.1021/acs.jnatprod.1c01211 (PMC8961869; doi:10.1021/acs.jnatprod.1c01211)
Supplement: Supplementary file 1 — np1c01211_si_002.pdf [file np1c01211_si_002.pdf]

Supporting Information

Cytotoxic Sesquiterpenoids from *Ammoides atlantica* Aerial Parts

Sihem Boudermine,[†,‡,•] Valentina Parisi,[§,•] Redouane Lemoui,[†] Tarek Boudiar,[⊥] Maria Giovanna Chini,[‖] Silvia Franceschelli,[§] Michela Pecoraro,[§] Maria Pascale,[§] Giuseppe Bifulco,[§] Alessandra Braca,[▽,○] Nunziatina De Tommasi,[*,§] and Marinella De Leo[▽,○]

[†]Département de Chimie, Université de Constantine 1, 25000 Constantine, Algeria

[‡]Département de Chimie, Université de 20 Aout 1955, 21000 Skikda, Algeria

[§]Dipartimento di Farmacia, Università degli Studi di Salerno, 84084 Fisciano (SA), Italy

[⊥] Biotechnology Research Center, 25000 Constantine, Algeria

[‖] Dipartimento di Bioscienze e Territorio, 86090 Pesche (IS), Italy

[▽]Dipartimento di Farmacia, Università di Pisa, 56126 Pisa, Italy

°CISUP, Centro per l'Integrazione della Strumentazione Scientifica, Università di Pisa, 56126 Pisa, Italy

• Sihem Boudermine and Valentina Parisi contributed equally to this work.

* Corresponding Author

Phone: +39-089-969754. Fax: +39-089-969602. E-mail: detommasi@unisa.it

Table of Contents

Figure S1. ^{1}H NMR spectrum of compound **1** (CD_3OD, 600 MHz)

Figure S2. ^{13}C NMR spectrum of compound **1** (CD_3OD, 150 MHz)

Figure S3. 1D TOCSY spectrum of compound **1** (CD_3OD, 600 MHz)

Figure S4. 1D TOCSY spectrum of compound **1** (CD_3OD, 600 MHz)

Figure S5. COSY spectrum of compound **1** (CD_3OD, 600 MHz)

Figure S6. HSQC spectrum of compound **1** (CD_3OD, 600 MHz)

Figure S7. HMBC spectrum of compound **1** (CD_3OD, 600 MHz)

Figure S8. HRESIMS of compound **1**

Figure S9. ^{1}H NMR spectrum of compound **2** (CD_3OD, 600 MHz)

Figure S10. 1D TOCSY spectrum of compound **2** (CD_3OD, 600 MHz)

Figure S11. COSY spectrum of compound **2** (CD_3OD, 600 MHz)

Figure S12. HSQC spectrum of compound **2** (CD_3OD, 600 MHz)

Figure S13. HMBC spectrum of compound **2** (CD_3OD, 600 MHz)

Figure S14. HRESIMS of compound **2**

Figure S15. ^{1}H NMR spectrum of compound **3** (CD_3OD, 600 MHz)

Figure S16. ^{13}C NMR spectrum of compound **3** (CD_3OD, 150 MHz)

Figure S17. COSY spectrum of compound **3** (CD_3OD, 600 MHz)

Figure S18. HSQC spectrum of compound **3** (CD_3OD, 600 MHz)

Figure S19. HMBC spectrum of compound **3** (CD_3OD, 600 MHz)

Figure S20. HRESIMS of compound **3**

Figure S21. ^{1}H NMR spectrum of compound **4** (CD_3OD, 600 MHz)

Figure S22. ^{13}C NMR spectrum of compound **4** (CD_3OD, 150 MHz)

Figure S23. COSY spectrum of compound **4** (CD_3OD, 600 MHz)

Figure S24. HSQC spectrum of compound **4** (CD_3OD, 600 MHz)

Figure S25. HMBC spectrum of compound **4** (CD_3OD, 600 MHz)

Figure S26. HRESIMS of compound **4**

Figure S27. ^{1}H NMR spectrum of compound **5** (CD_3OD, 600 MHz)

Figure S28. ^{13}C NMR spectrum of compound **5** (CD_3OD, 150 MHz)

Figure S29. COSY spectrum of compound **5** (CD_3OD, 600 MHz)

Figure S30. HSQC spectrum of compound **5** (CD_3OD, 600 MHz)

Figure S31. HMBC spectrum of compound **5** (CD_3OD, 600 MHz)

Figure S32. HRESIMS of compound **5**

Figure S33. ^{1}H NMR spectrum of compound **6** (CD_3OD, 600 MHz)

Figure S34. ^{13}C NMR spectrum of compound **6** (CD_3OD, 150 MHz)

Figure S35. COSY spectrum of compound **6** (CD_3OD, 600 MHz)

Figure S36. HSQC spectrum of compound **6** (CD_3OD, 600 MHz)

Figure S37. HMBC spectrum of compound **6** (CD_3OD, 600 MHz)

Figure S38. HRESIMS of compound **6**

Figure S39. ^{1}H NMR spectrum of compound **7** (CD_3OD, 600 MHz)

Figure S40. ^{13}C NMR spectrum of compound **7** (CD_3OD, 150 MHz)

Figure S41. HSQC spectrum of compound **7** (CD_3OD, 600 MHz)

Figure S42. HMBC spectrum of compound **7** (CD_3OD, 600 MHz)

Figure S43. HRESIMS of compound **7**

Table S1. Full MS and MS/MS data registered in negative ionization mode of compounds detected in the chloroform extract of *Ammoides atlantica* aerial parts

Table S2. Quantitative amounts (g/100 g ± SD of DW) of constituents isolated from *Ammoides atlantica* aerial parts.

Table S3. ^{13}C experimental and calculated NMR chemical shifts for **1**, with $^{a}|\Delta\delta|(^{13}C)$ and ^{b}MAE values

Table S4. ^{1}H experimental and calculated NMR chemical shifts for **1,** with $^{a}|\Delta\delta|(^{1}H)$ and ^{b}MAE values

Table S5. ^{13}C experimental and calculated NMR chemical shifts for **2**, with $^{a}|\Delta\delta|(^{13}C)$ and ^{b}MAE values

Table S6. ^{1}H experimental and calculated NMR chemical shifts for **2**, with $^{a}|\Delta\delta|(^{1}H)$ and ^{b}MAE values

Table S7. ^{13}C experimental and calculated NMR chemical shifts for **6,** with $^{a}|\Delta\delta|(^{13}C)$ and ^{b}MAE values

Table S8. ^{1}H experimental and calculated NMR chemical shifts for **6,** with $^{a}|\Delta\delta|(^{1}H)$ and ^{b}MAE values

Table S9. ^{13}C experimental and calculated NMR chemical shifts for **7,** with $^{a}|\Delta\delta|(^{13}C)$ and ^{b}MAE values

Table S10. ^{1}H experimental and calculated NMR chemical shifts for **7,** with $^{a}|\Delta\delta|(^{1}H)$ and ^{b}MAE values

Figure S1. ^{1}H NMR spectrum of compound **1** (CD_3OD, 600 MHz)

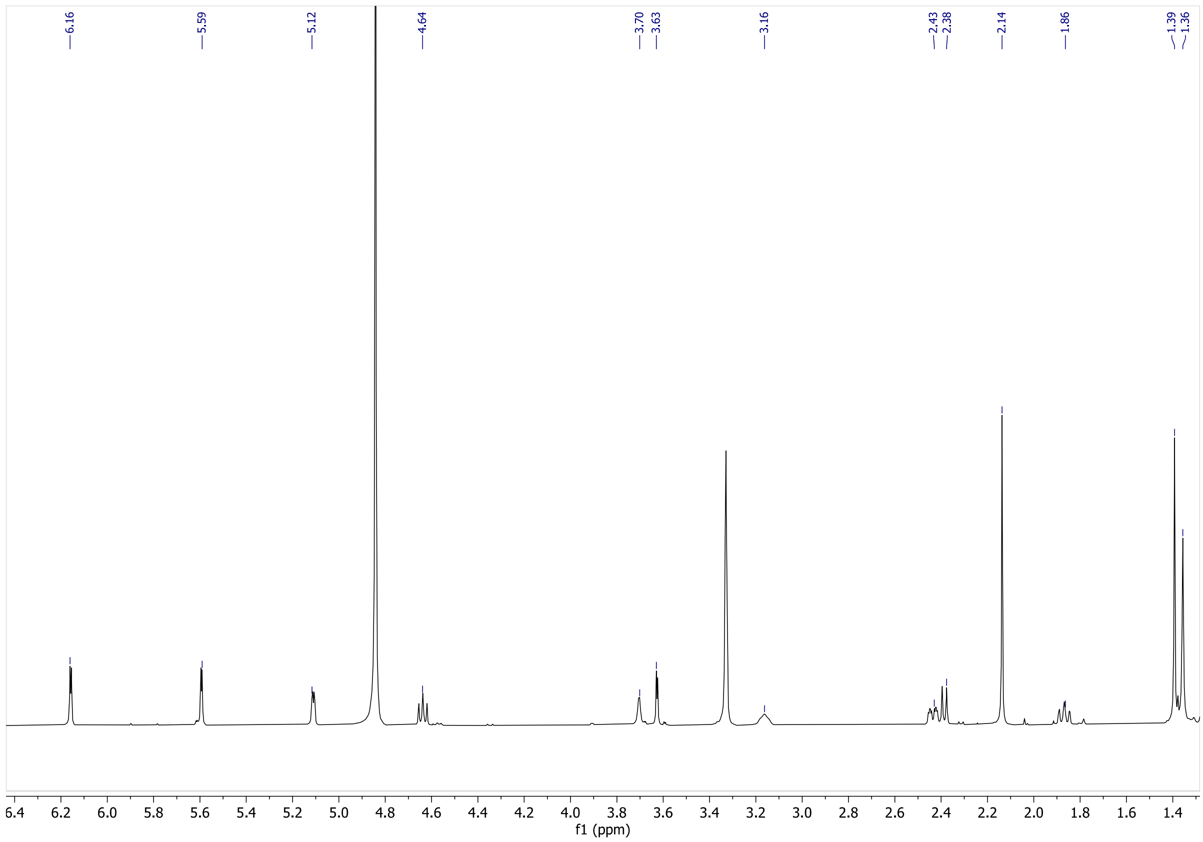

Figure S2. ^{13}C NMR spectrum of compound **1** (CD_3OD, 150 MHz)

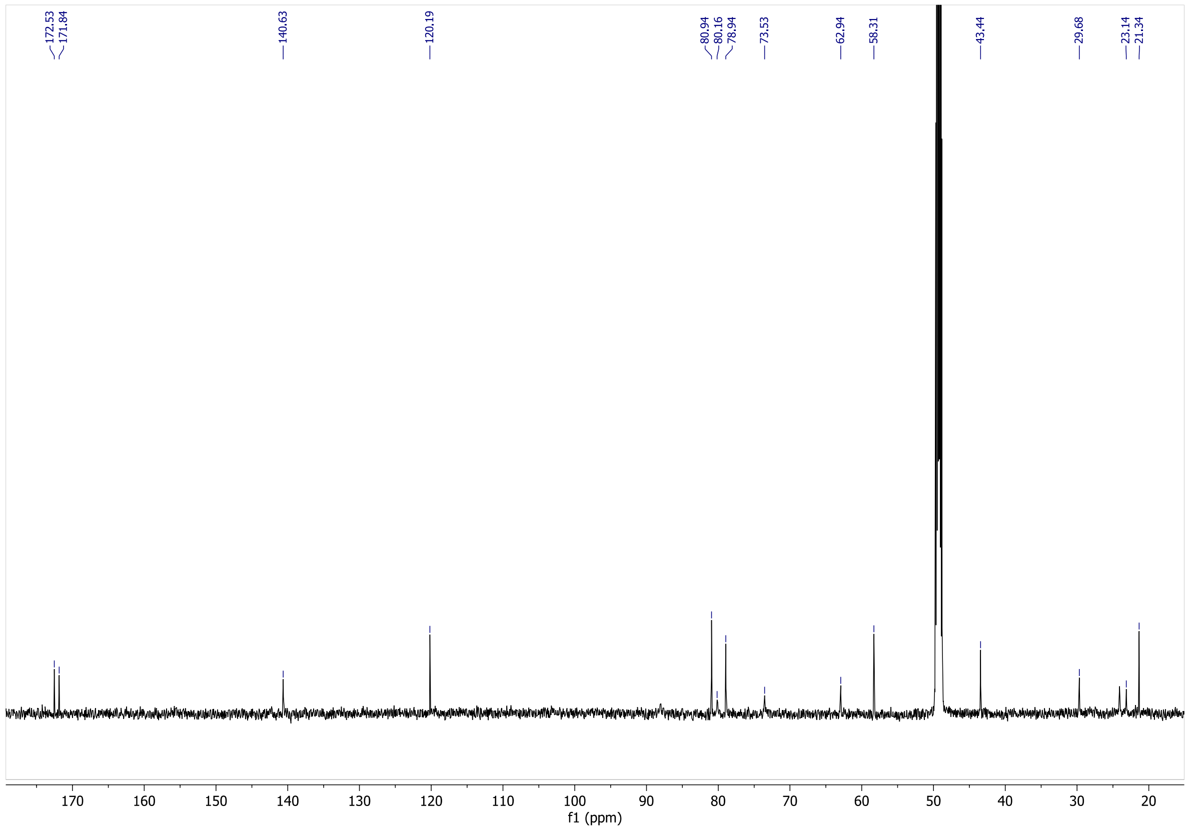

Figure S3. 1D TOCSY spectrum of compound **1** (CD_3OD, 600 MHz)

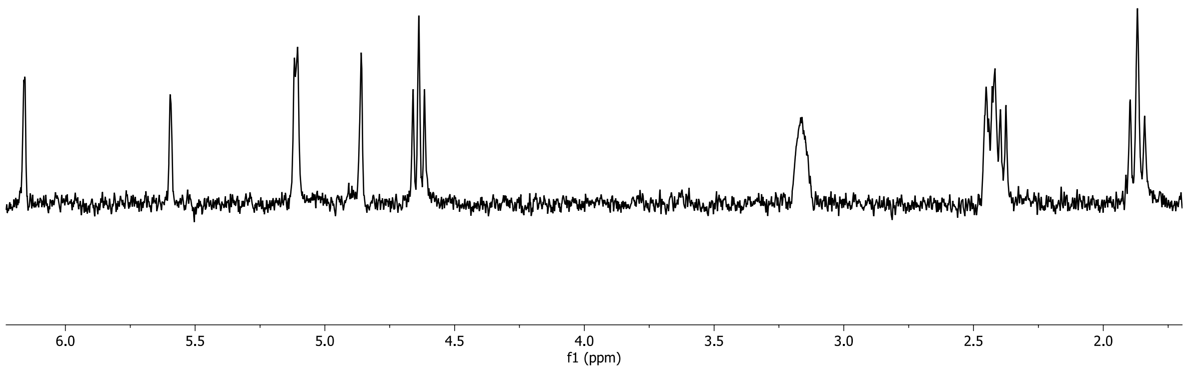

Figure S4. 1D TOCSY spectrum of compound **1** (CD_3OD, 600 MHz)

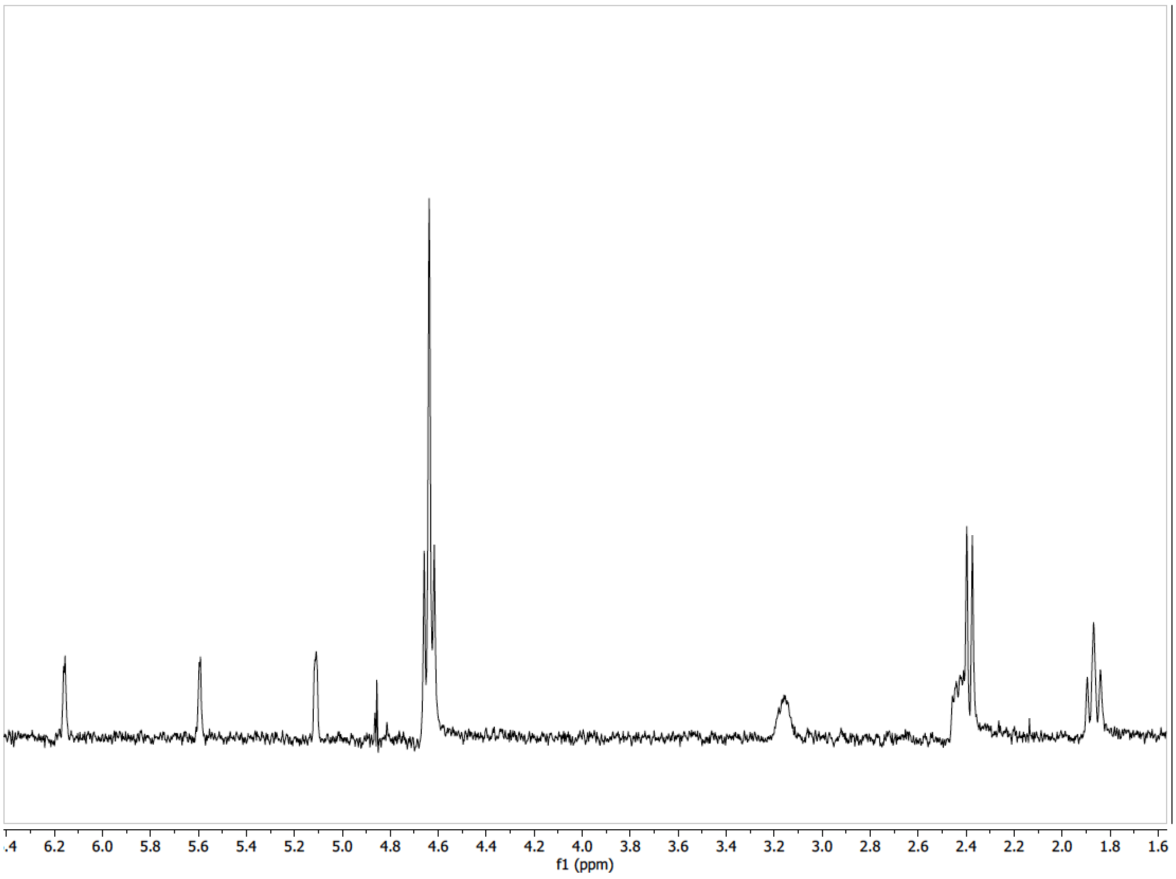

Figure S5. COSY spectrum of compound **1** (CD_3OD, 600 MHz)

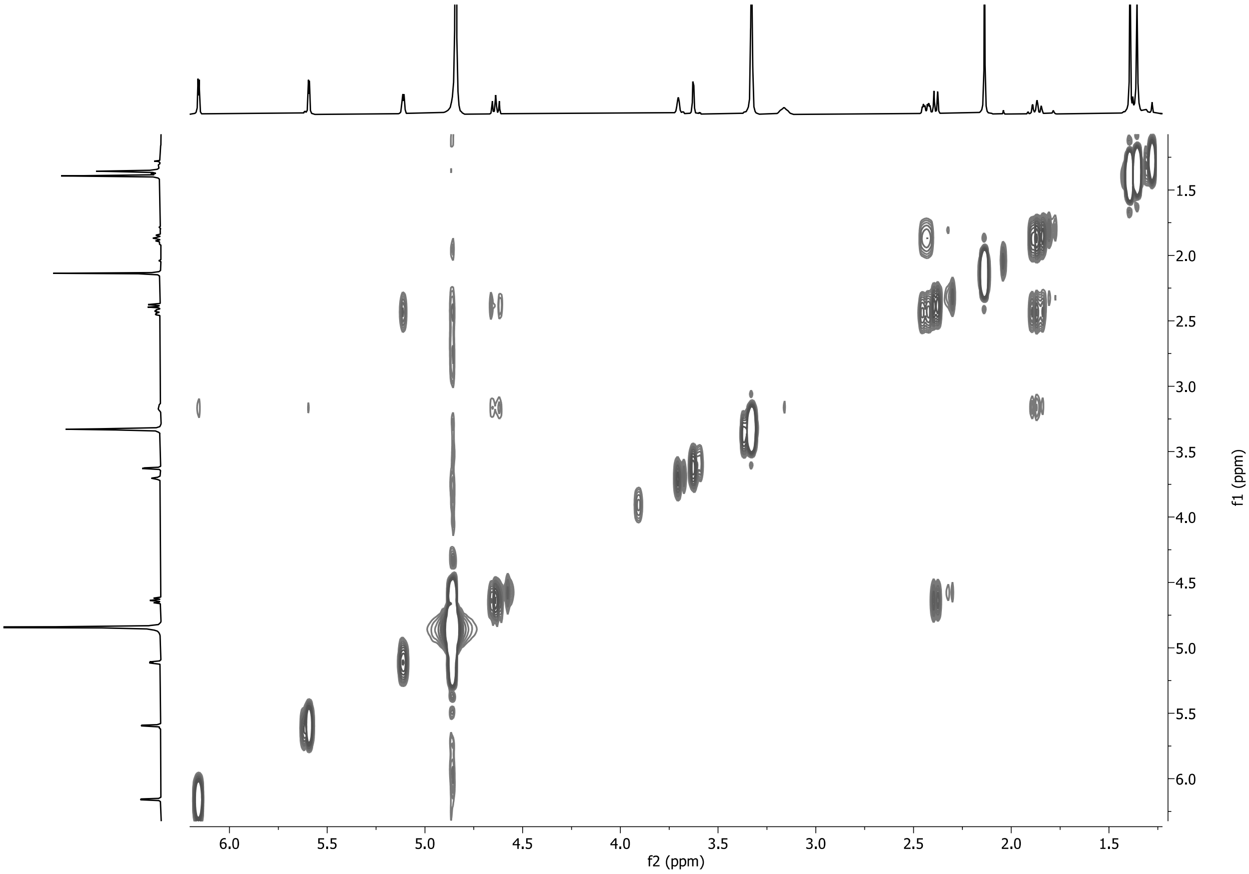

Figure S6. HSQC spectrum of compound **1** (CD_3OD, 600 MHz)

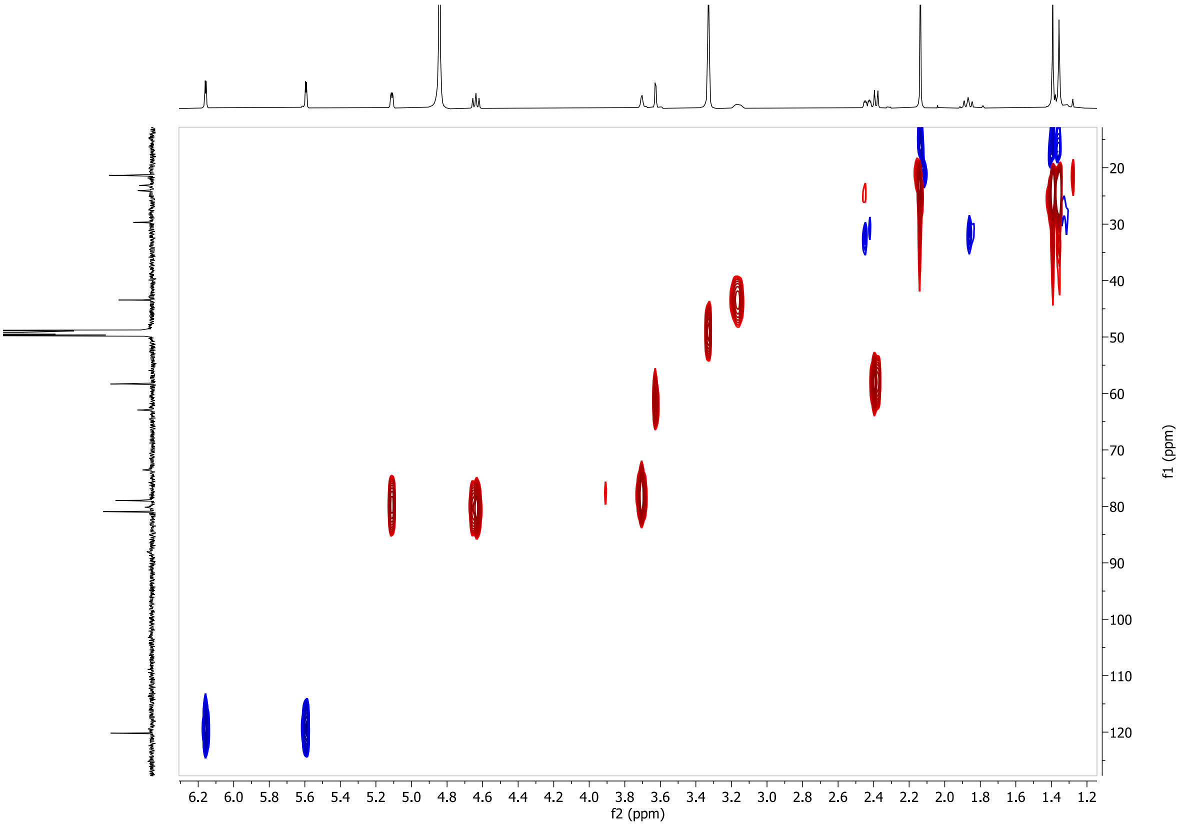

Figure S7. HMBC spectrum of compound **1** (CD_3OD, 600 MHz)

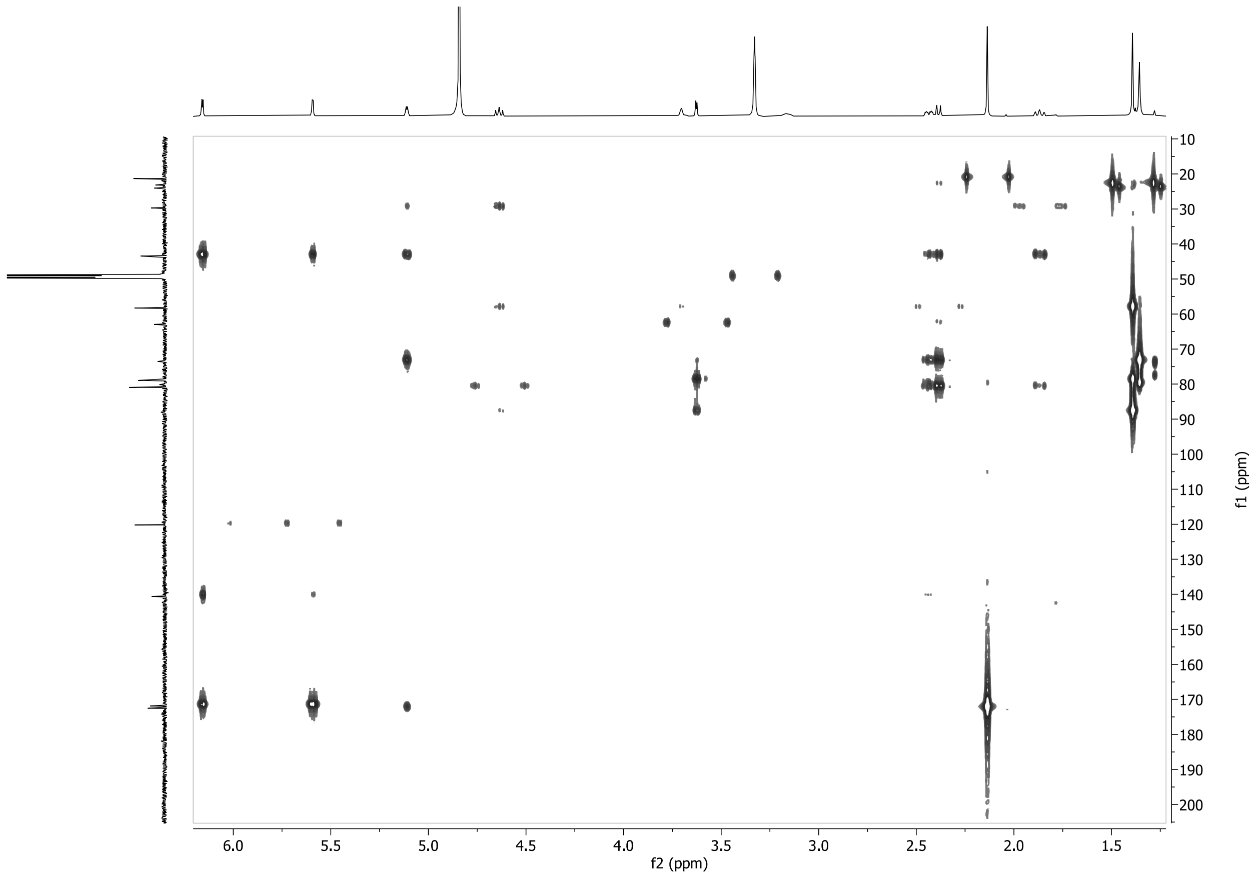

Figure S8. HRESIMS of compound **1**

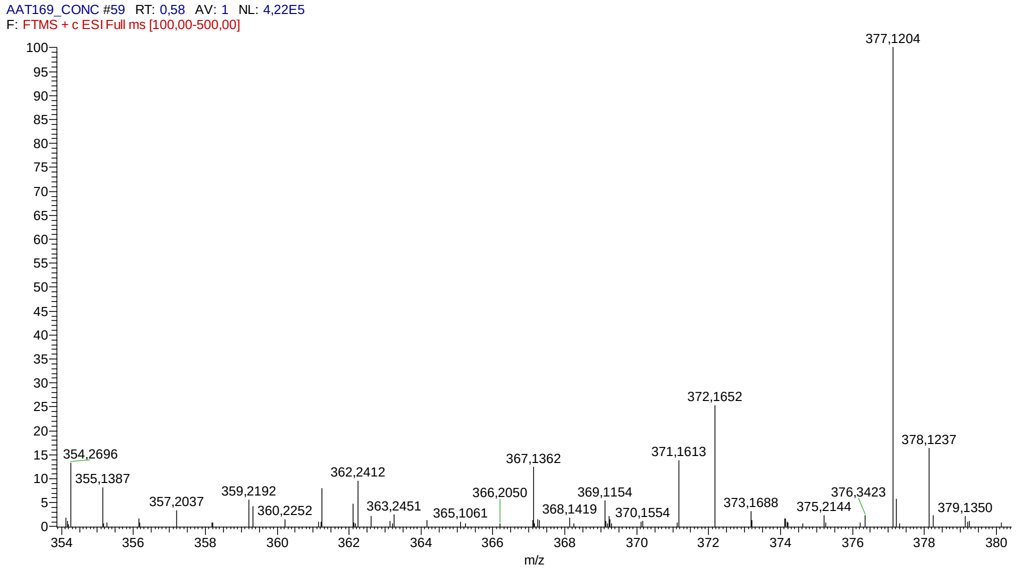

Figure S9. ^{1}H NMR spectrum of compound **2** (CD_3OD, 600 MHz)

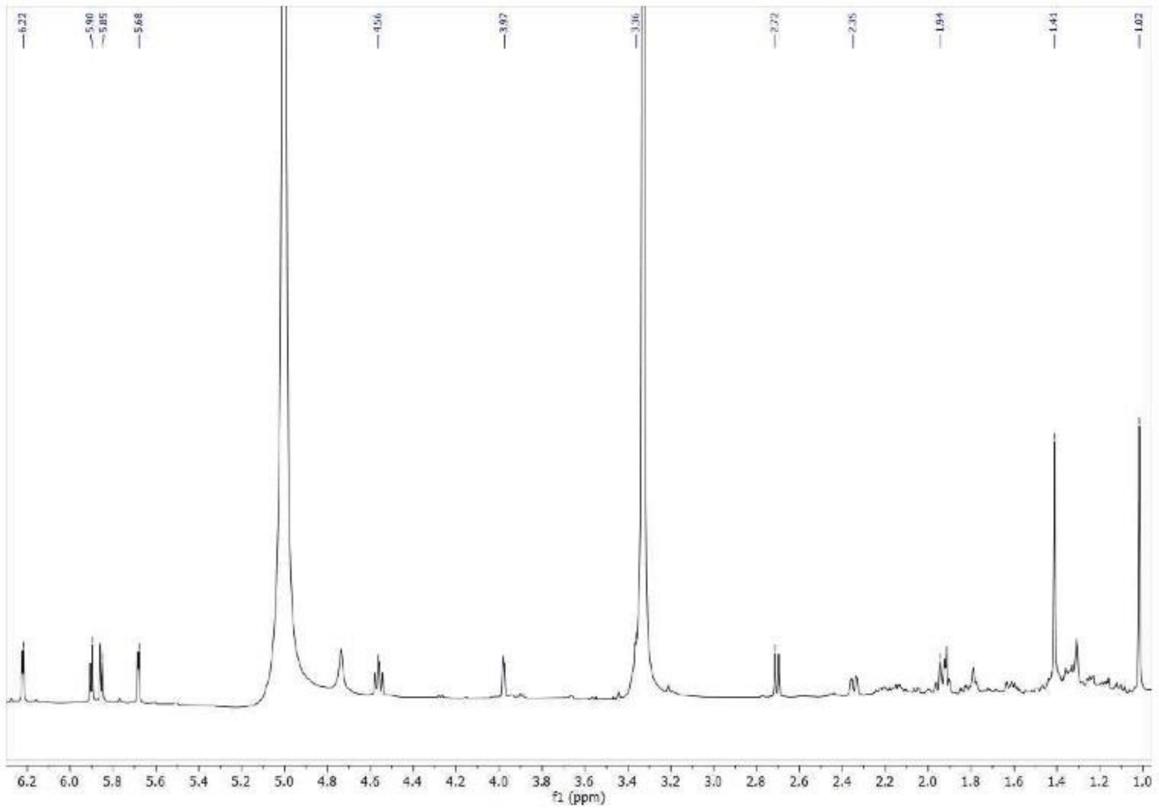

Figure S10. 1D TOCSY spectrum of compound **2** (CD_3OD, 600 MHz)

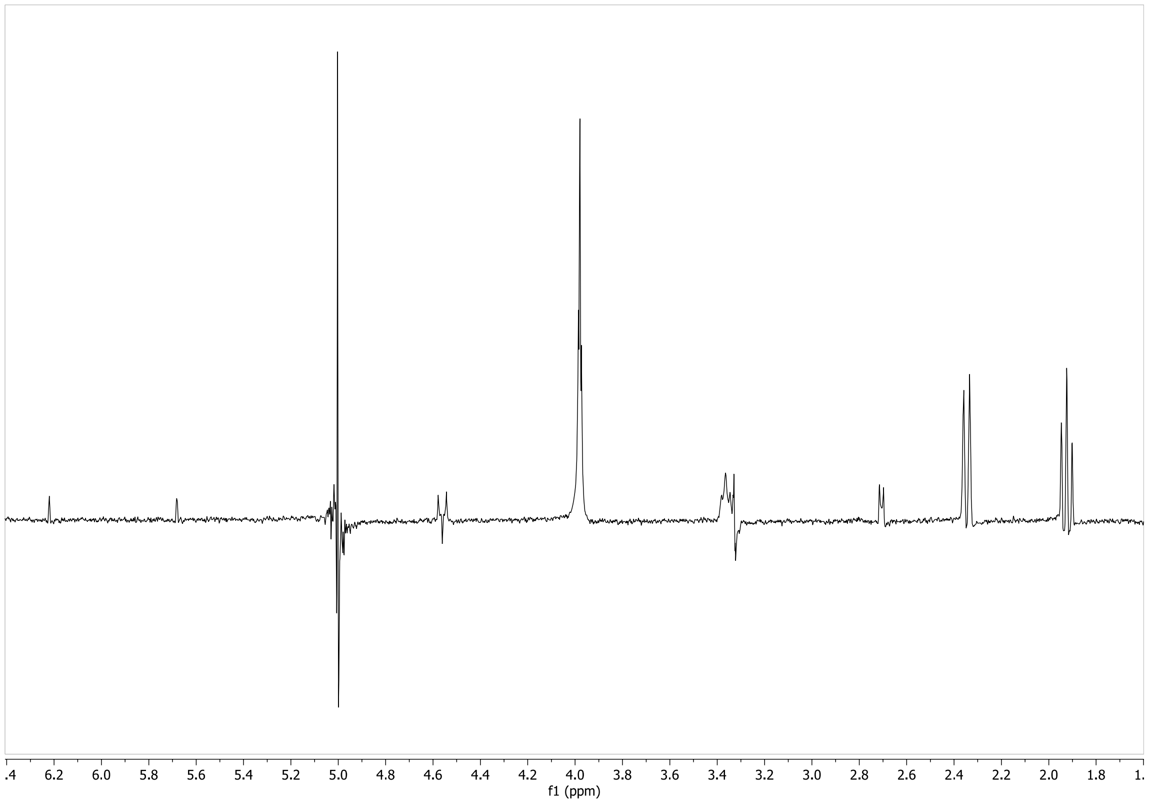

Figure S11. COSY spectrum of compound **2** (CD_3OD, 600 MHz)

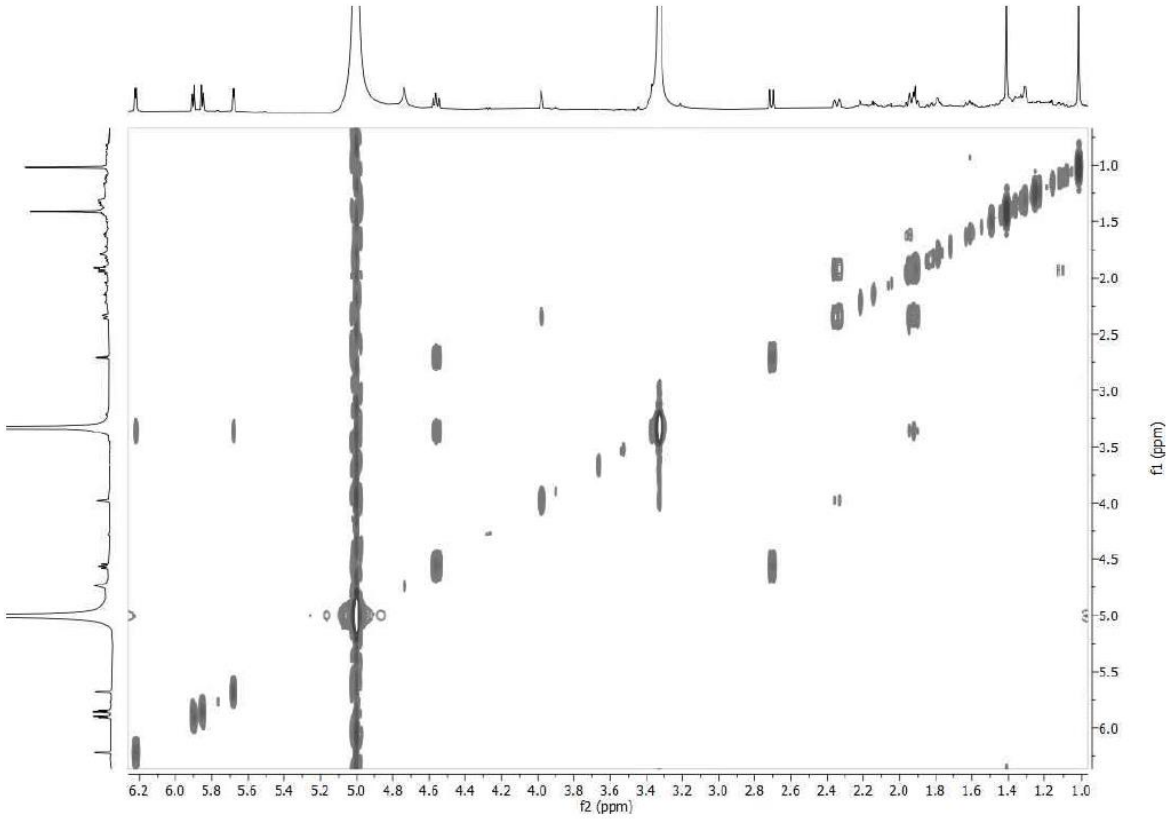

Figure S12. HSQC spectrum of compound **2** (CD_3OD, 600 MHz)

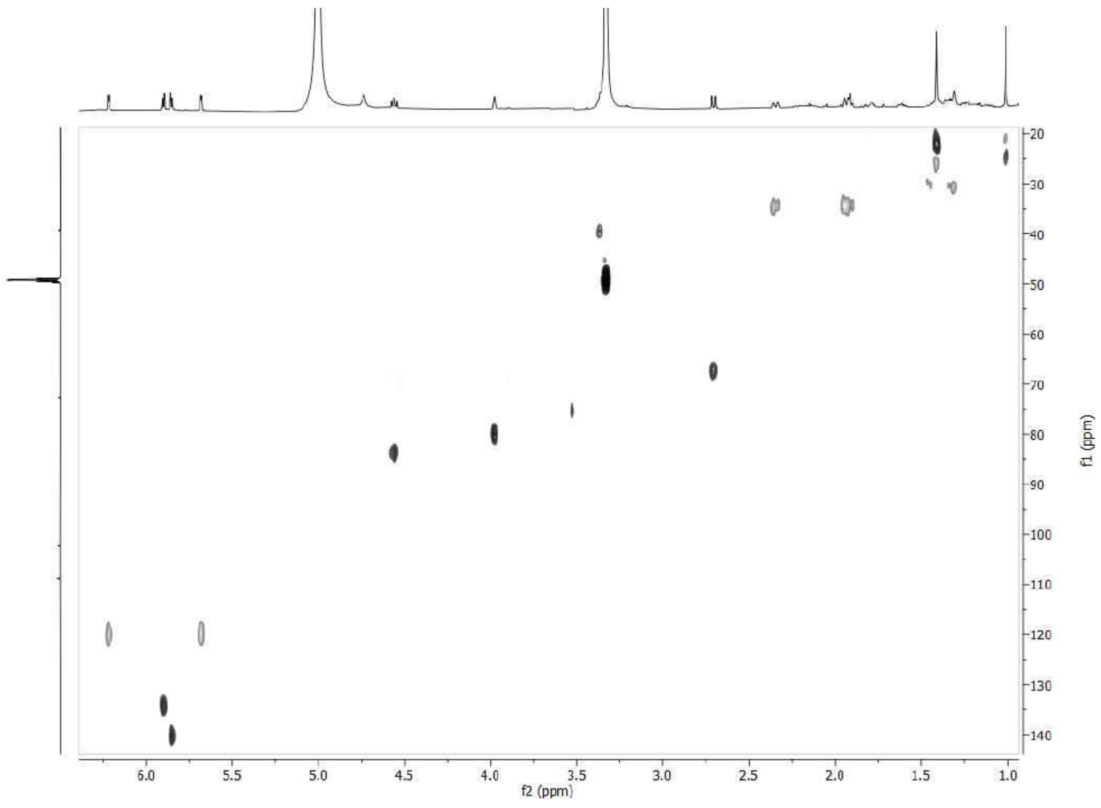

Figure S13. HMBC spectrum of compound **2** (CD_3OD, 600 MHz)

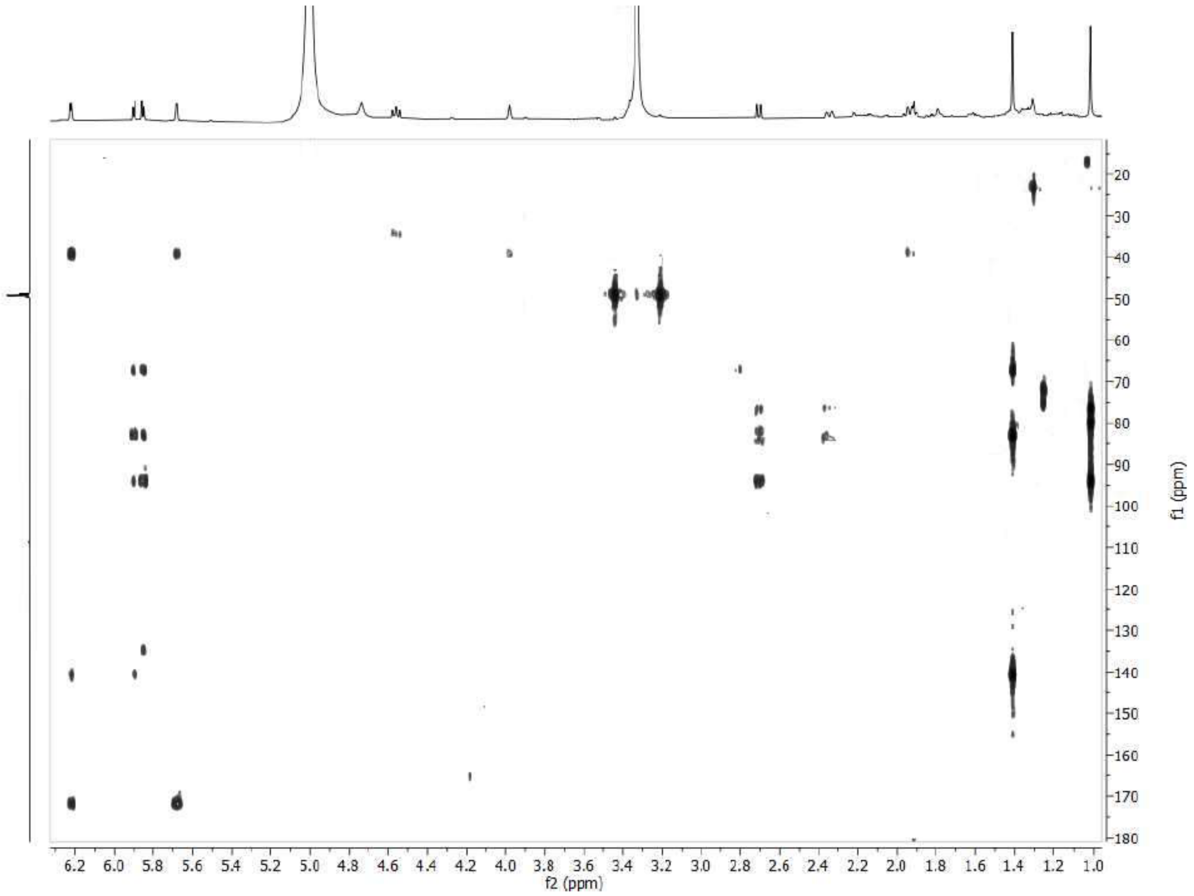

Figure S14. HRESIMS of compound **2**

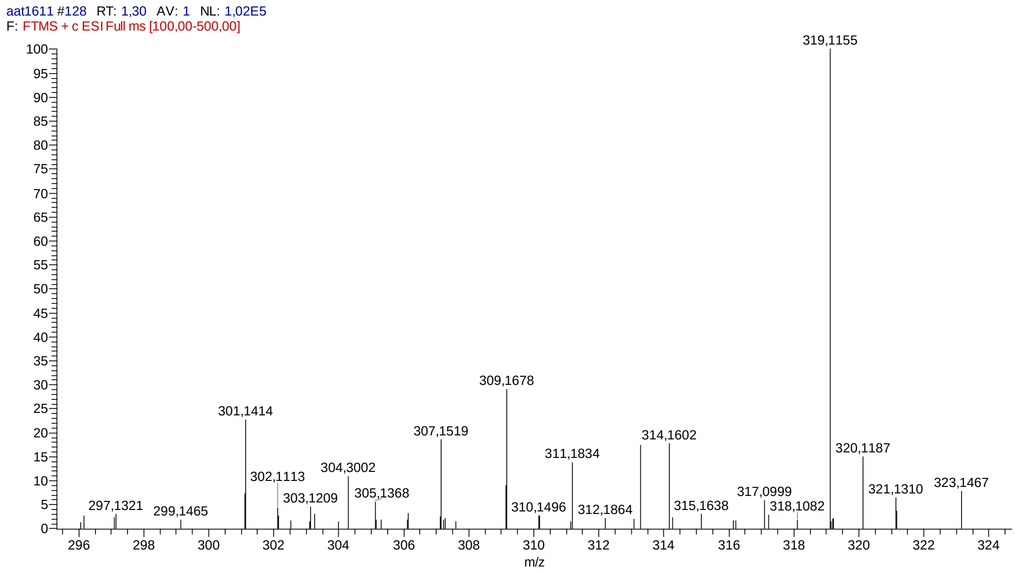

Figure S15. ^{1}H NMR spectrum of compound **3** (CD_3OD, 600 MHz)

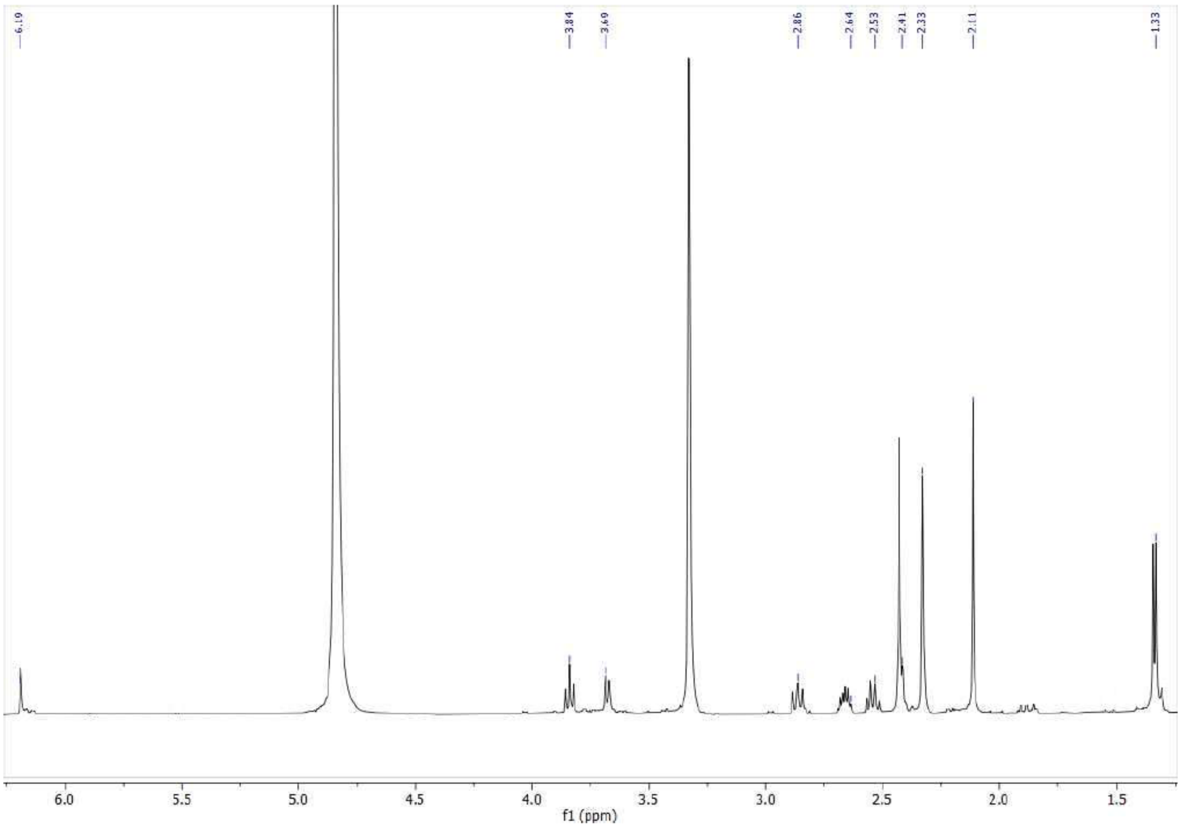

Figure S16. ^{13}C NMR spectrum of compound **3** (CD_3OD, 150 MHz)

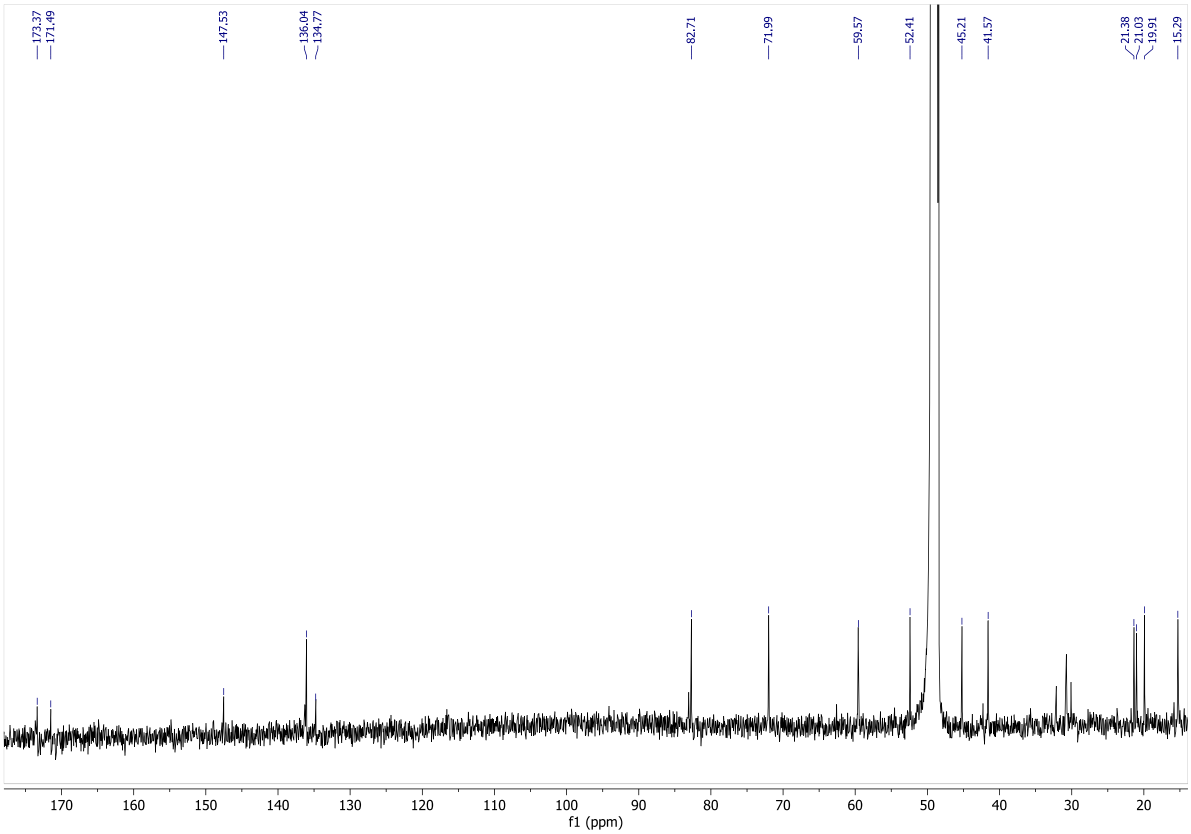

Figure S17. COSY spectrum of compound **3** (CD_3OD, 600 MHz)

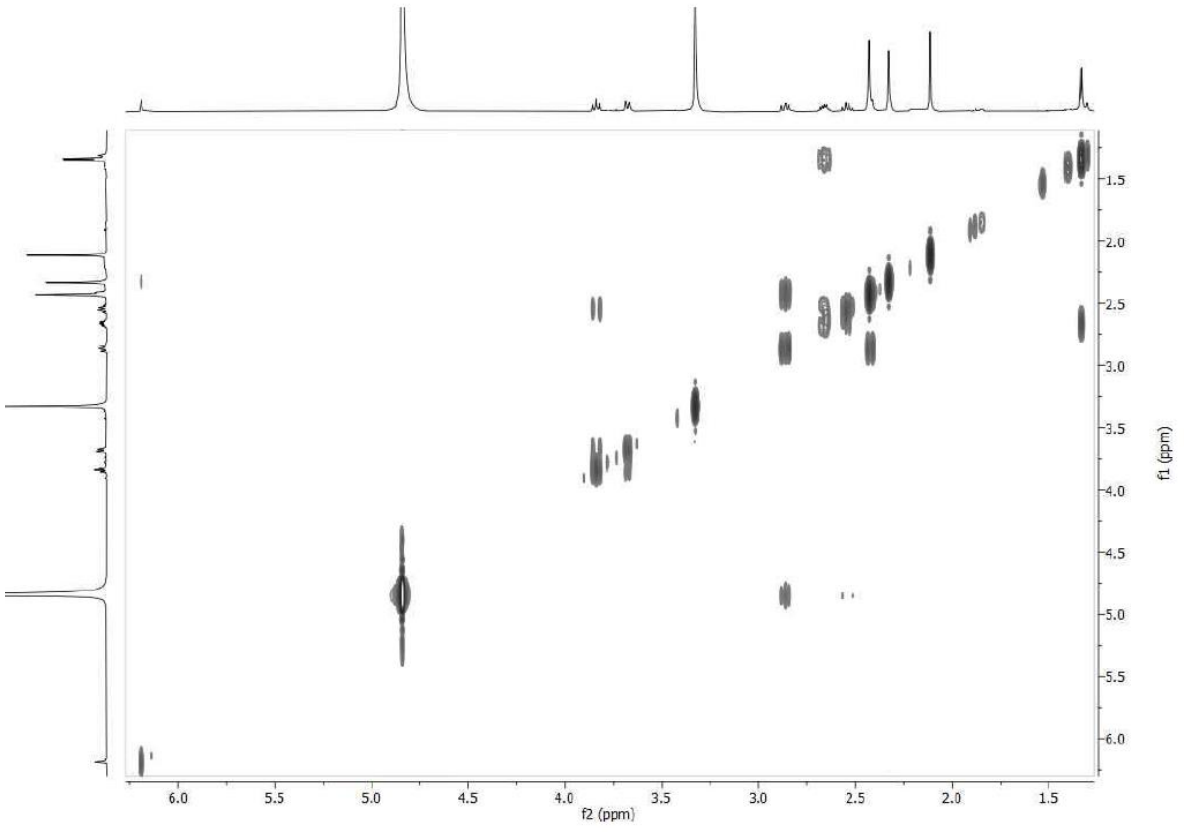

Figure S18. HSQC spectrum of compound **3** (CD_3OD, 600 MHz)

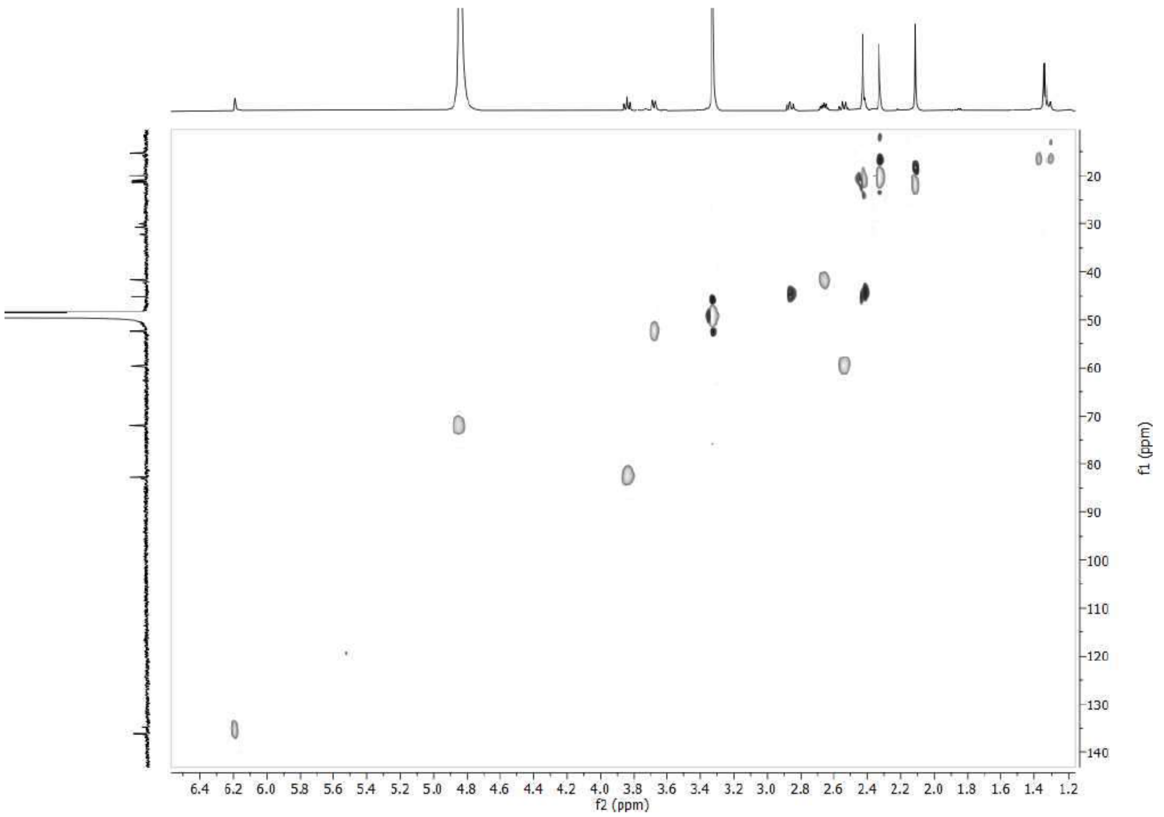

Figure S19. HMBC spectrum of compound **3** (CD_3OD, 600 MHz)

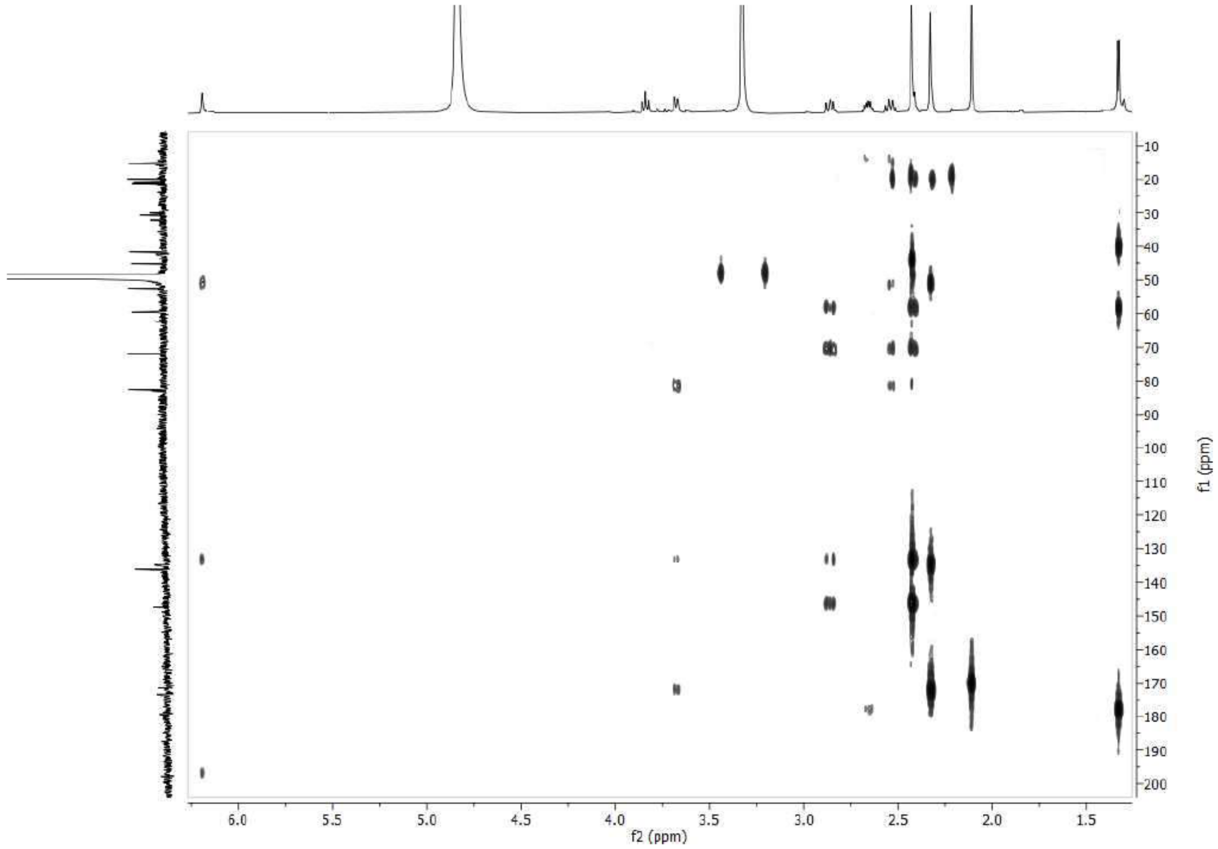

Figure S20. HRESIMS of compound **3**

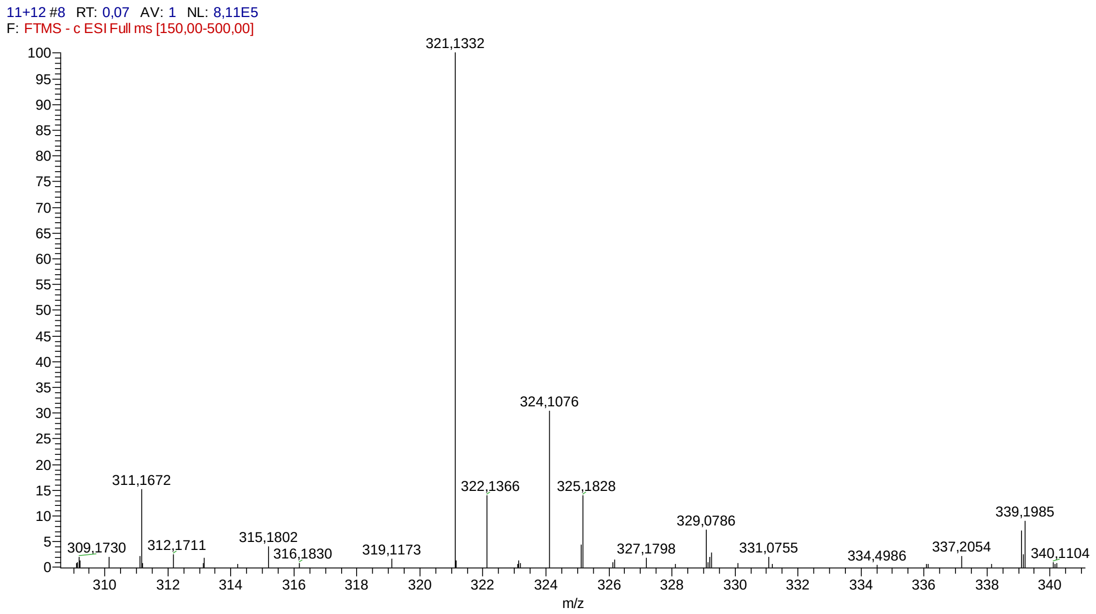

Figure S21. ^{1}H NMR spectrum of compound **4** (CD_3OD, 600 MHz)

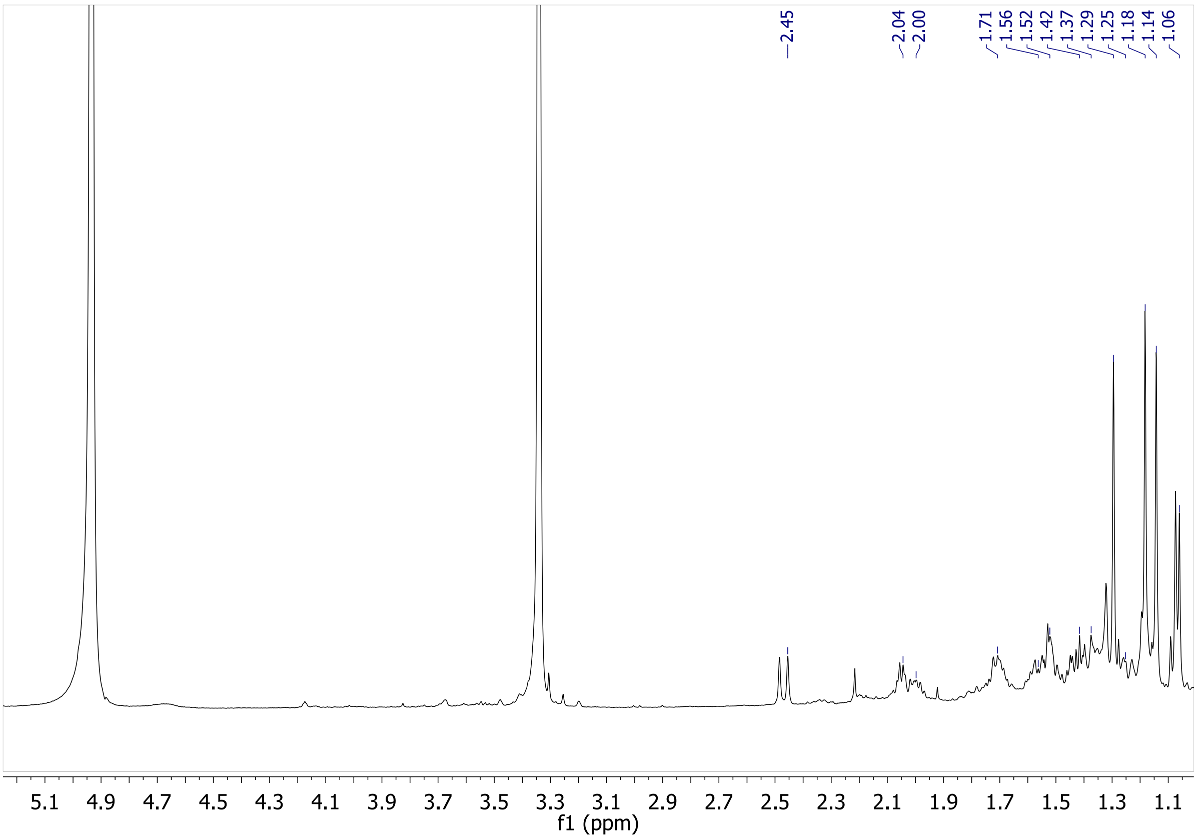

Figure S22. ^{13}C NMR spectrum of compound **4** (CD_3OD, 150 MHz)

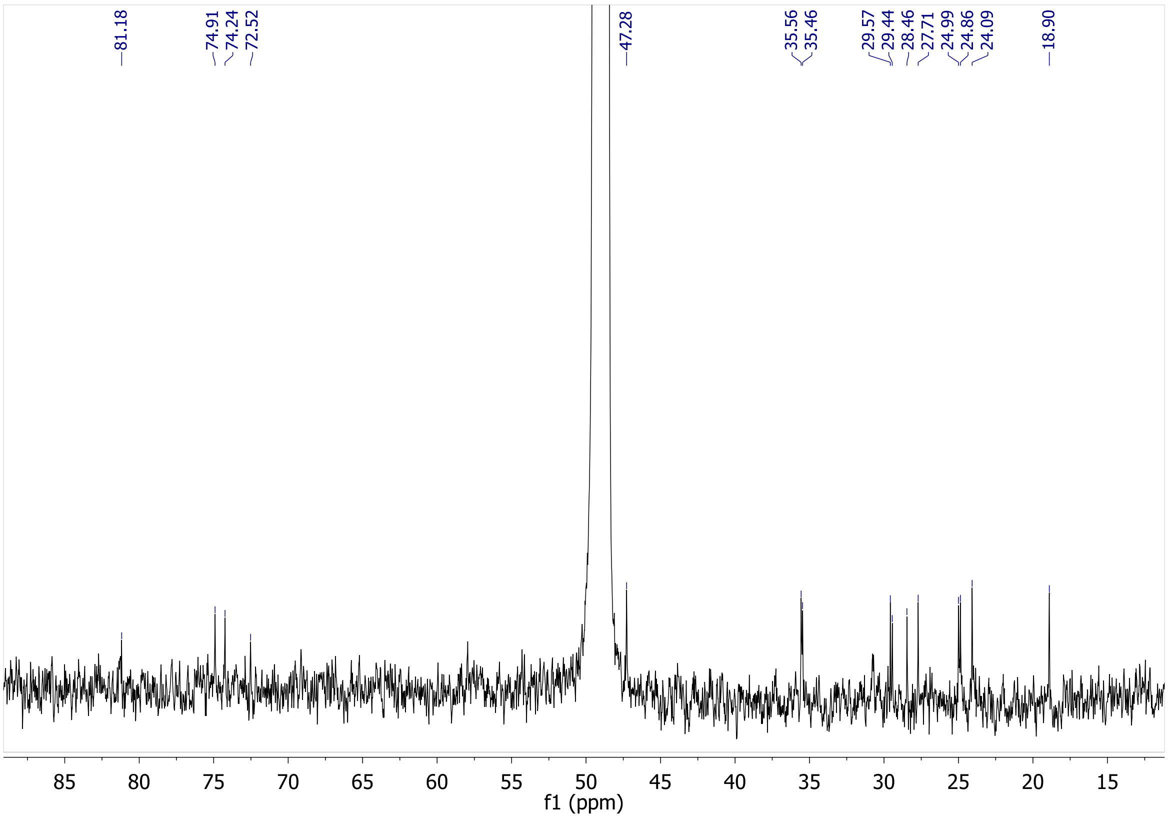

Figure S23. COSY spectrum of compound **4** (CD_3OD, 600 MHz)

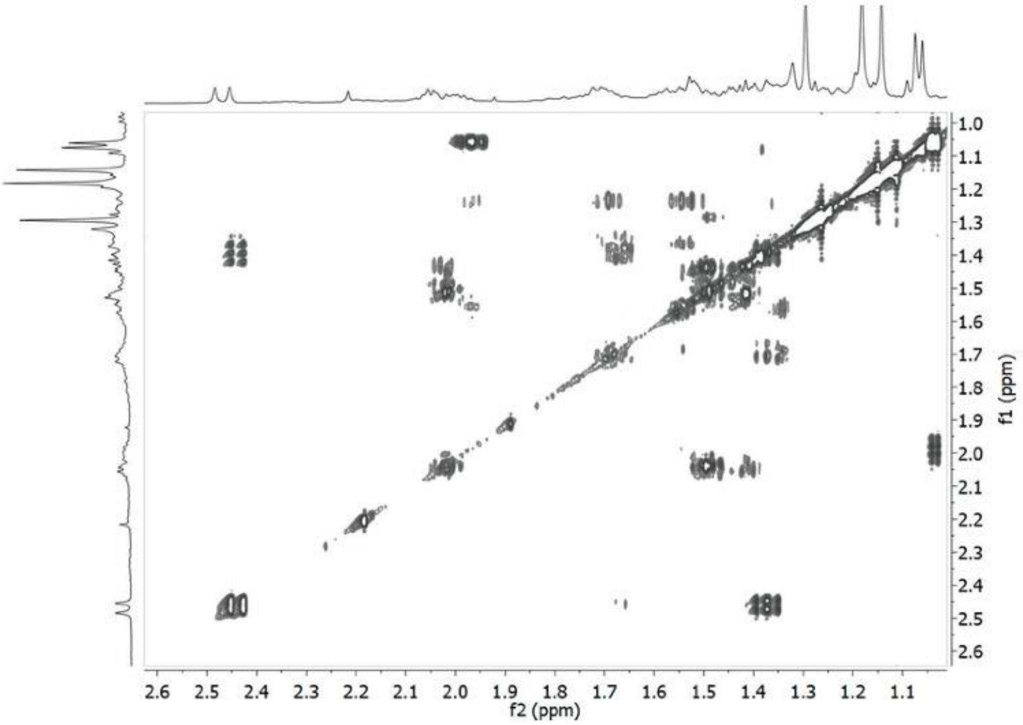

Figure S24. HSQC spectrum of compound **4** (CD_3OD, 600 MHz)

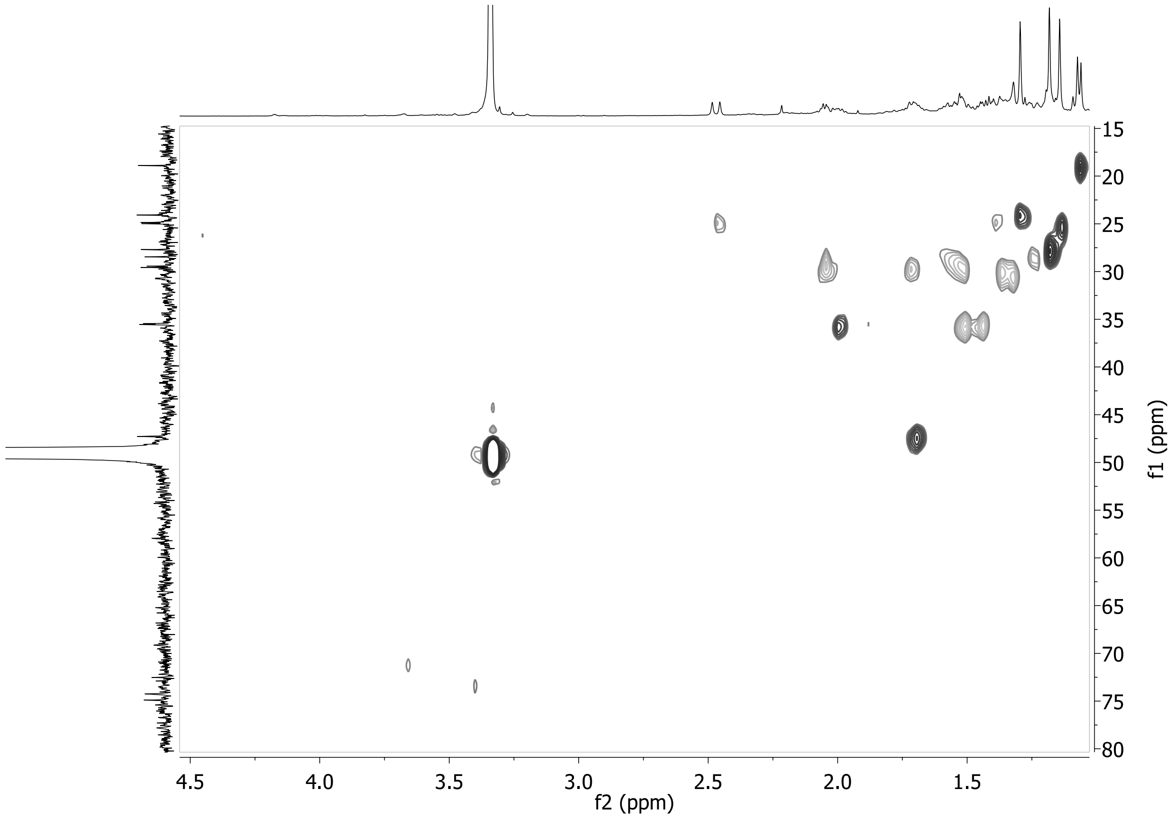

Figure S25. HMBC spectrum of compound **4** (CD_3OD, 600 MHz)

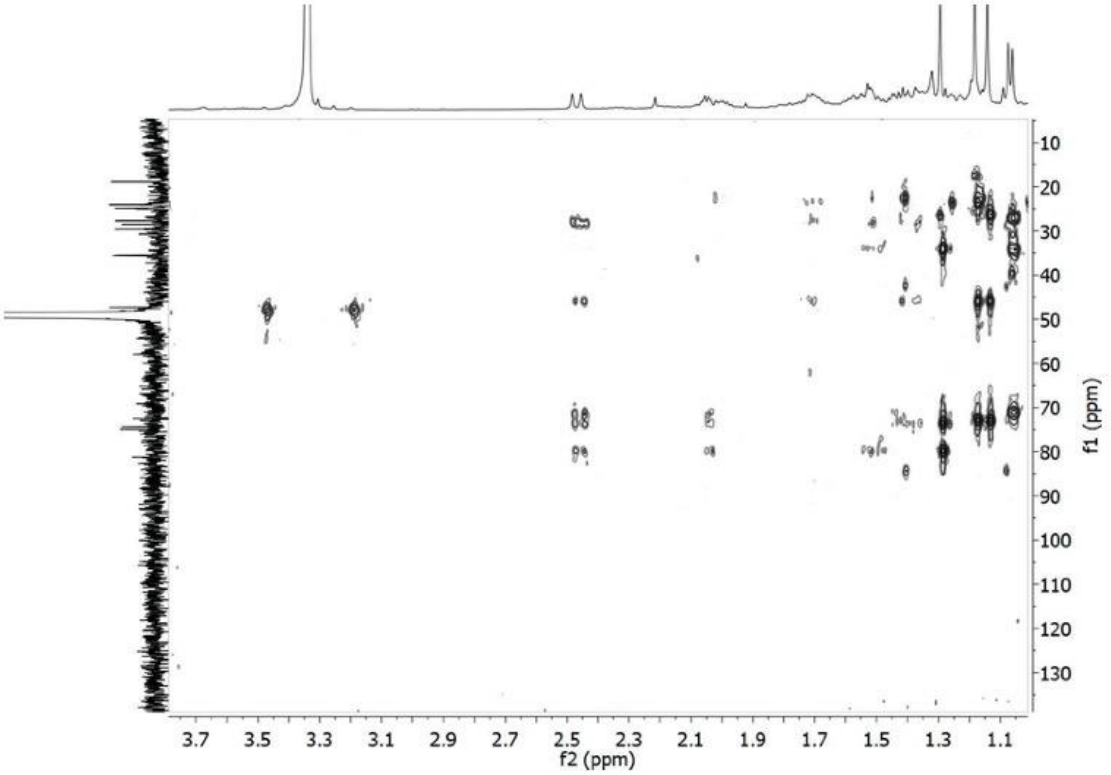

Figure S26. HRESIMS of compound **4**

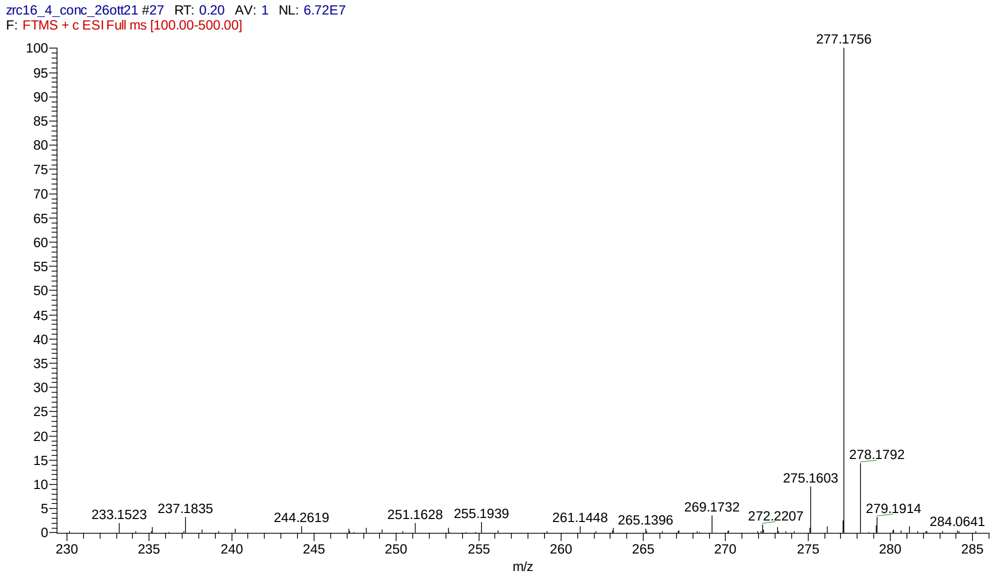

Figure S27. ^{1}H NMR spectrum of compound **5** (CD_3OD, 600 MHz)

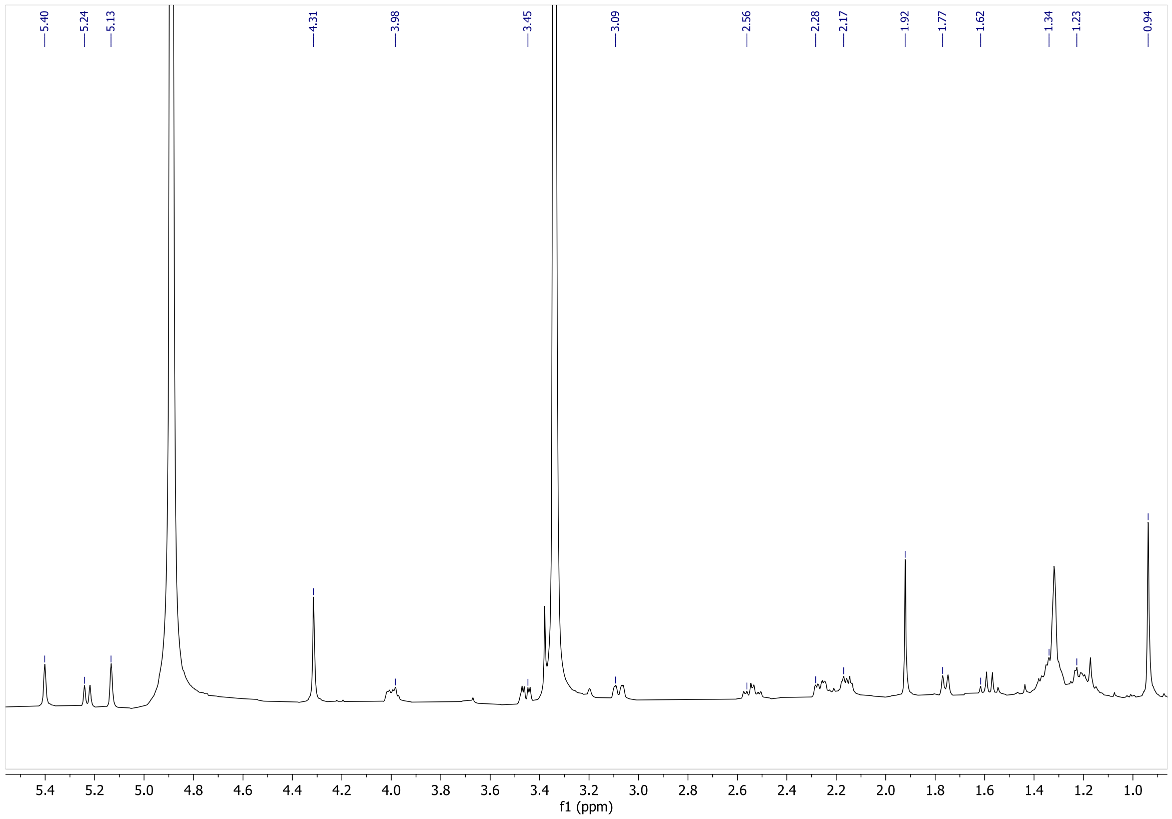

Figure S28. ^{13}C NMR spectrum of compound **5** (CD_3OD, 150 MHz)

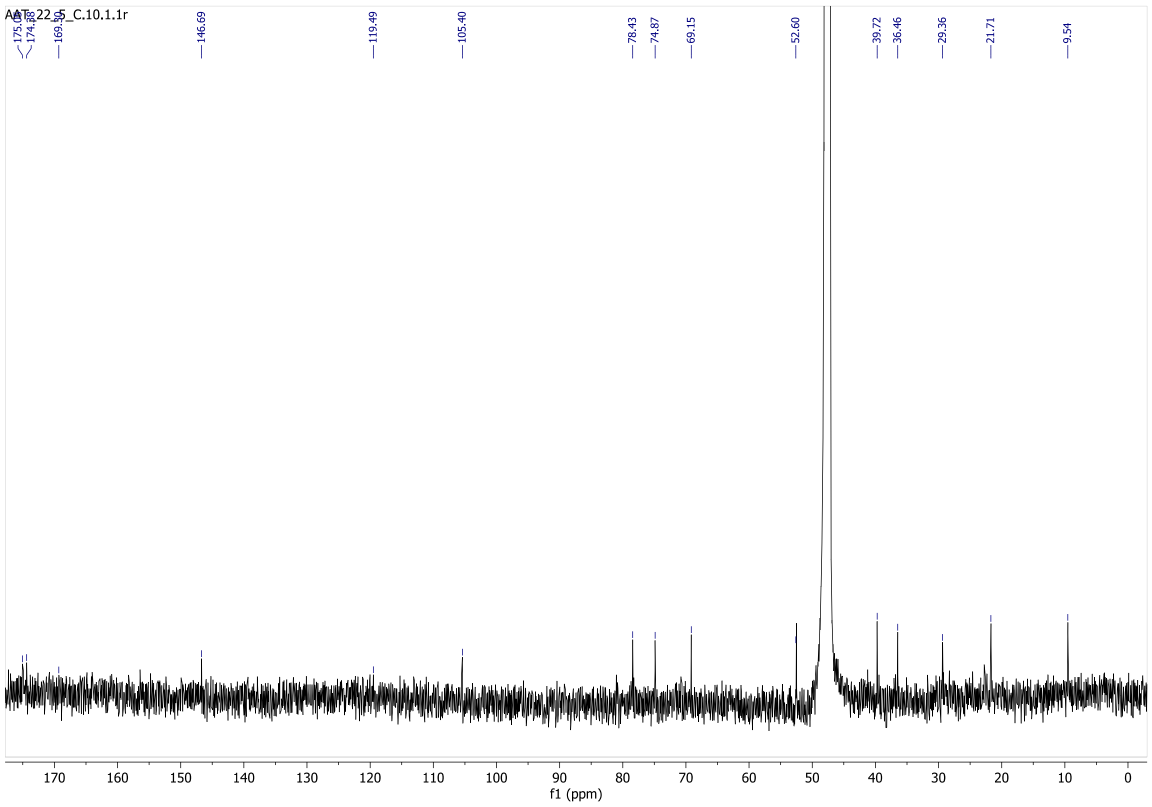

Figure S29. COSY spectrum of compound **5** (CD_3OD, 600 MHz)

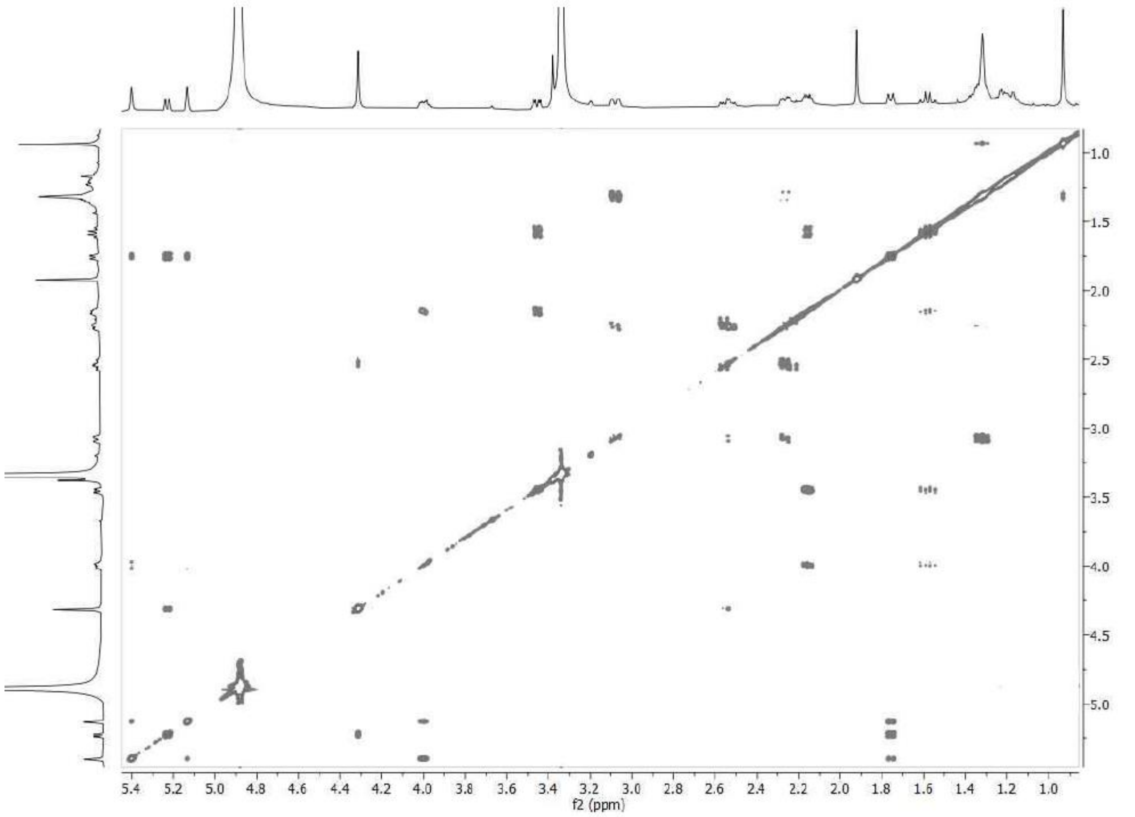

Figure S30. HSQC spectrum of compound **5** (CD_3OD, 600 MHz)

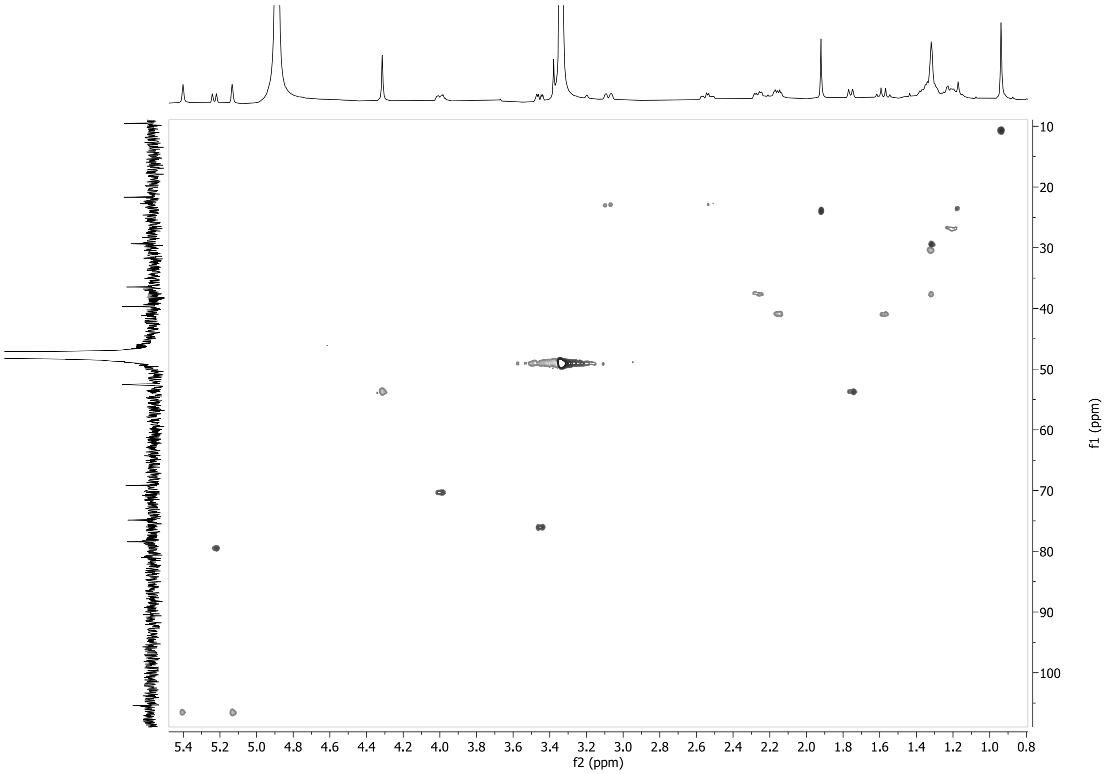

Figure S31. HMBC spectrum of compound **5** (CD_3OD, 600 MHz)

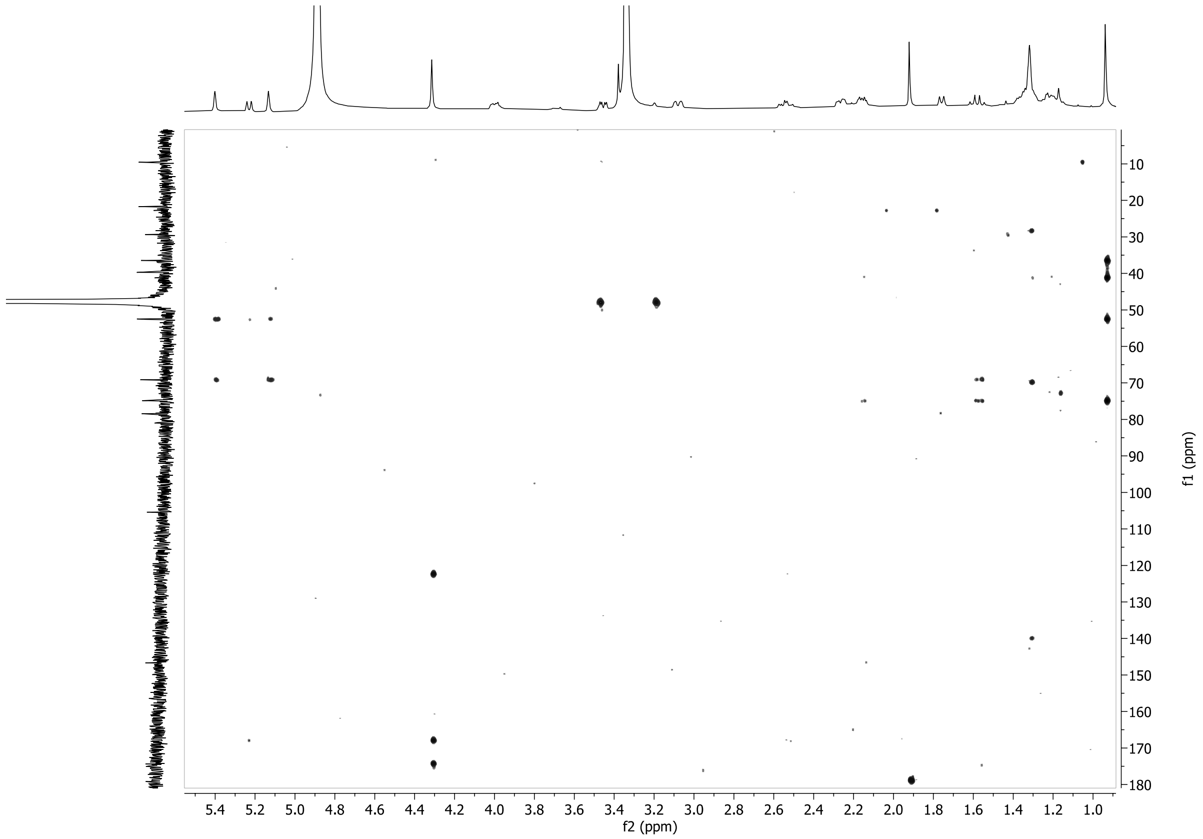

Figure S32. HRESIMS of compound **5**

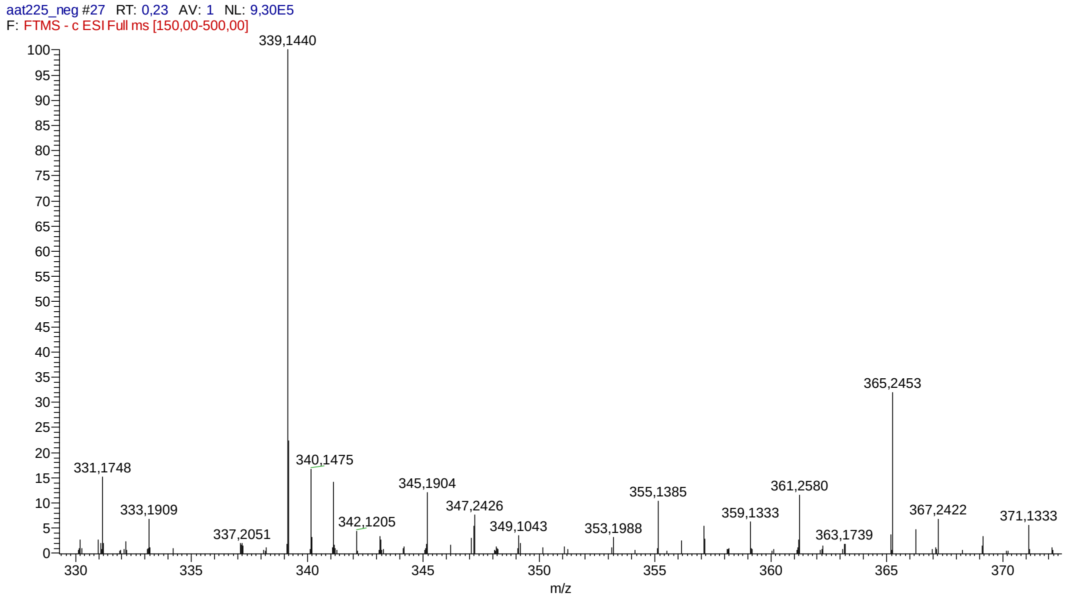

Figure S33. ^{1}H NMR spectrum of compound **6** (CD_3OD, 600 MHz)

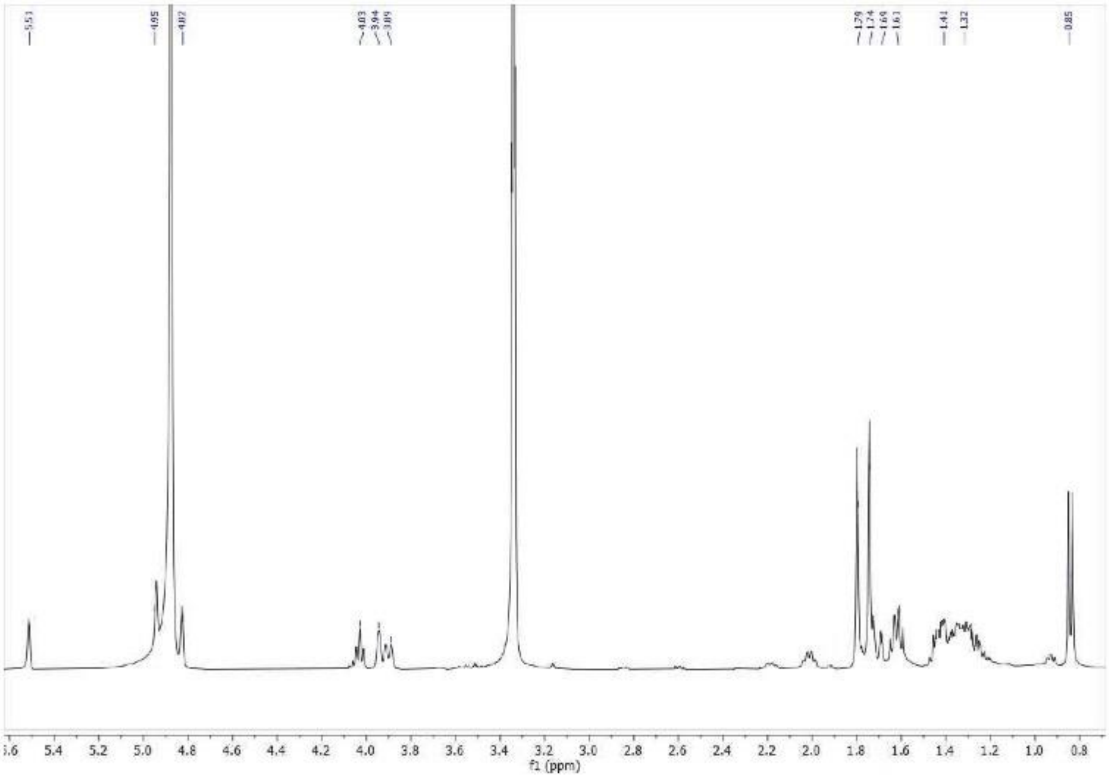

Figure S34. ^{13}C NMR spectrum of compound **6** (CD_3OD, 150 MHz)

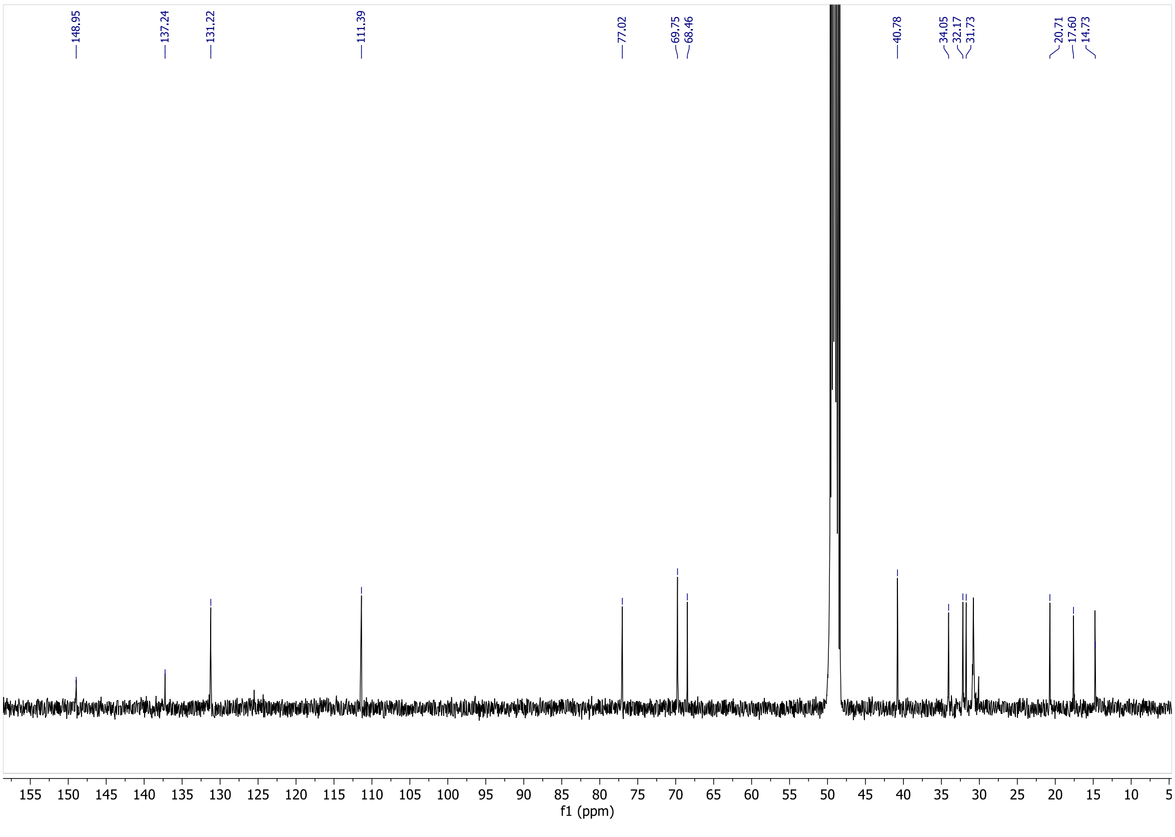

Figure S35. COSY spectrum of compound **6** (CD_3OD, 600 MHz)

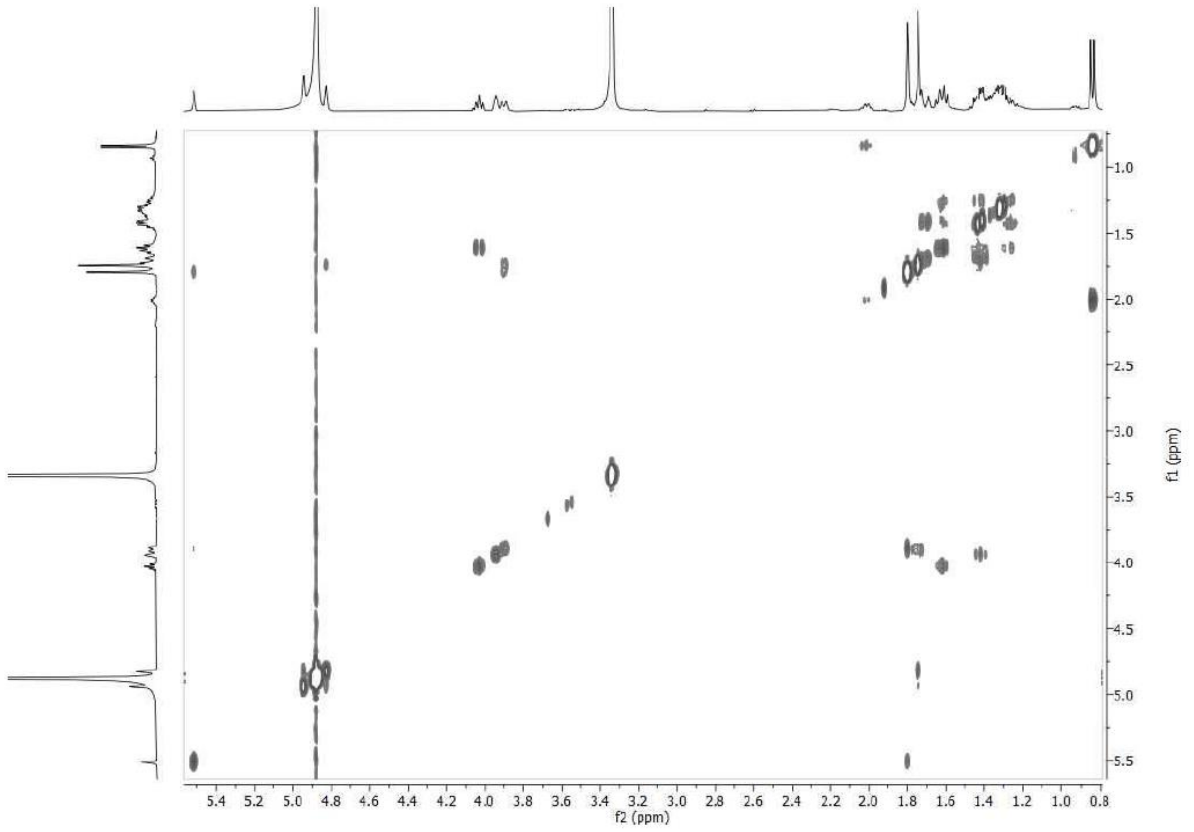

Figure S36. HSQC spectrum of compound **6** (CD_3OD, 600 MHz)

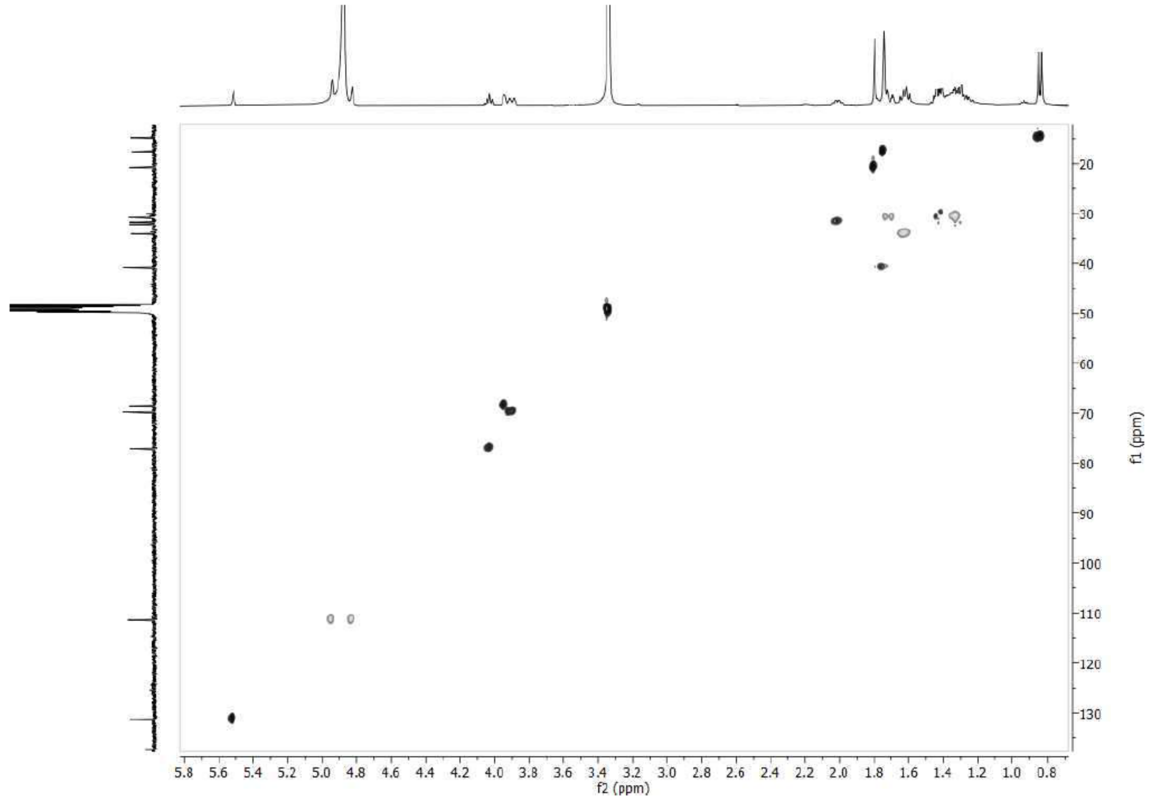

Figure S37. HMBC spectrum of compound **6** (CD_3OD, 600 MHz)

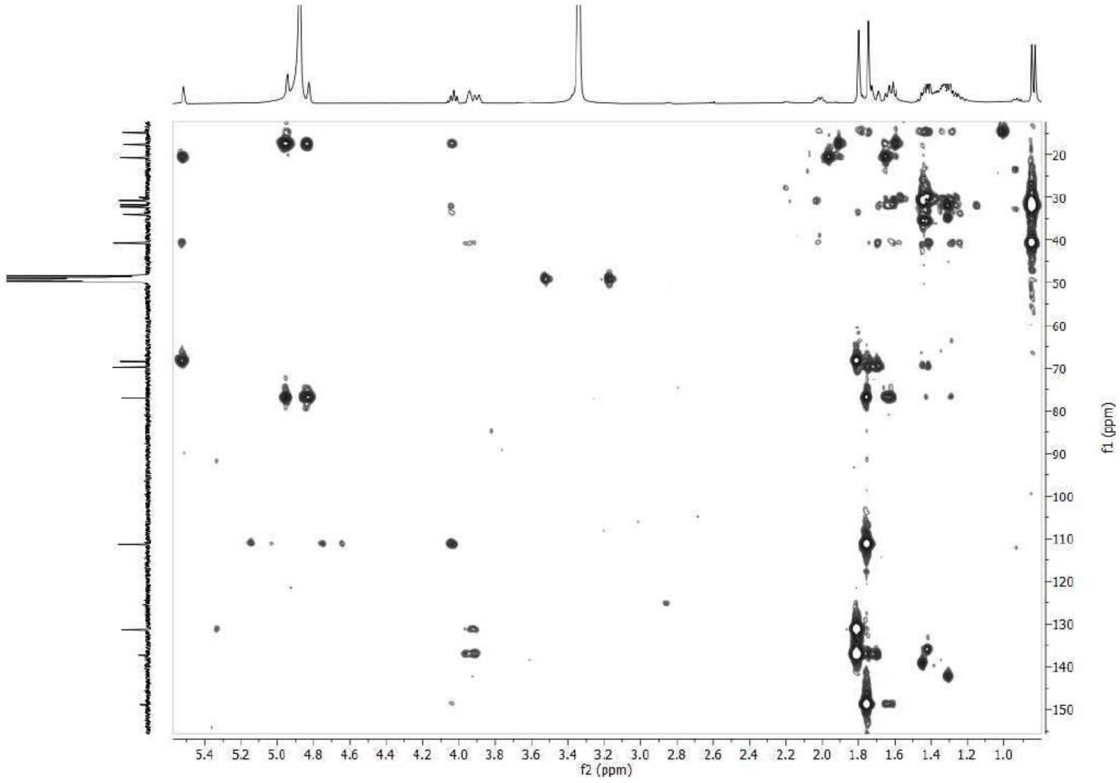

Figure S38. HRESIMS of compound **6**

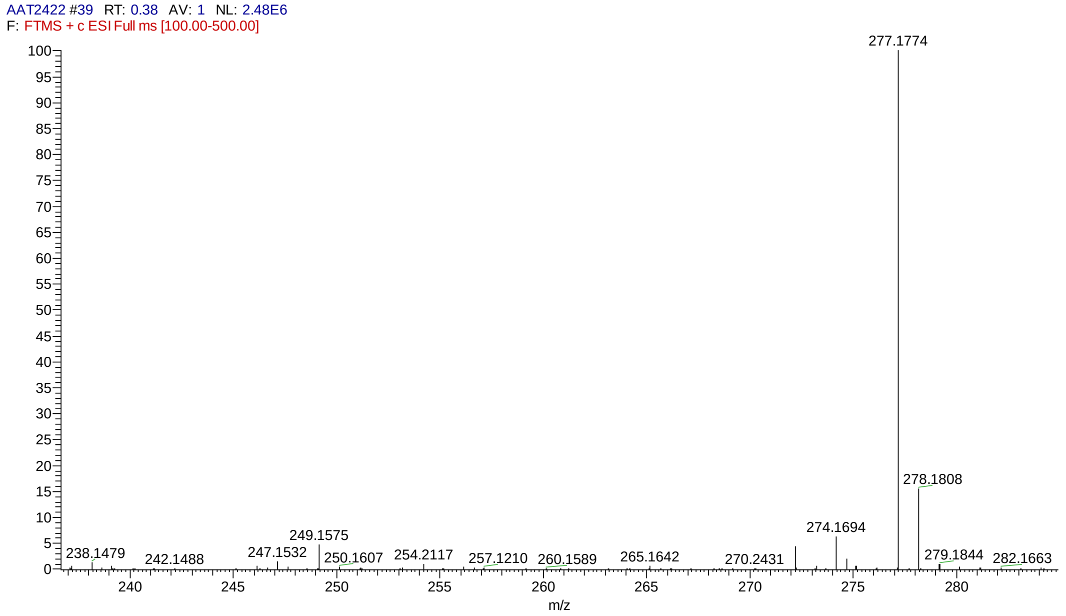

Figure S39. ^{1}H NMR spectrum of compound **7** (CD_3OD, 600 MHz)

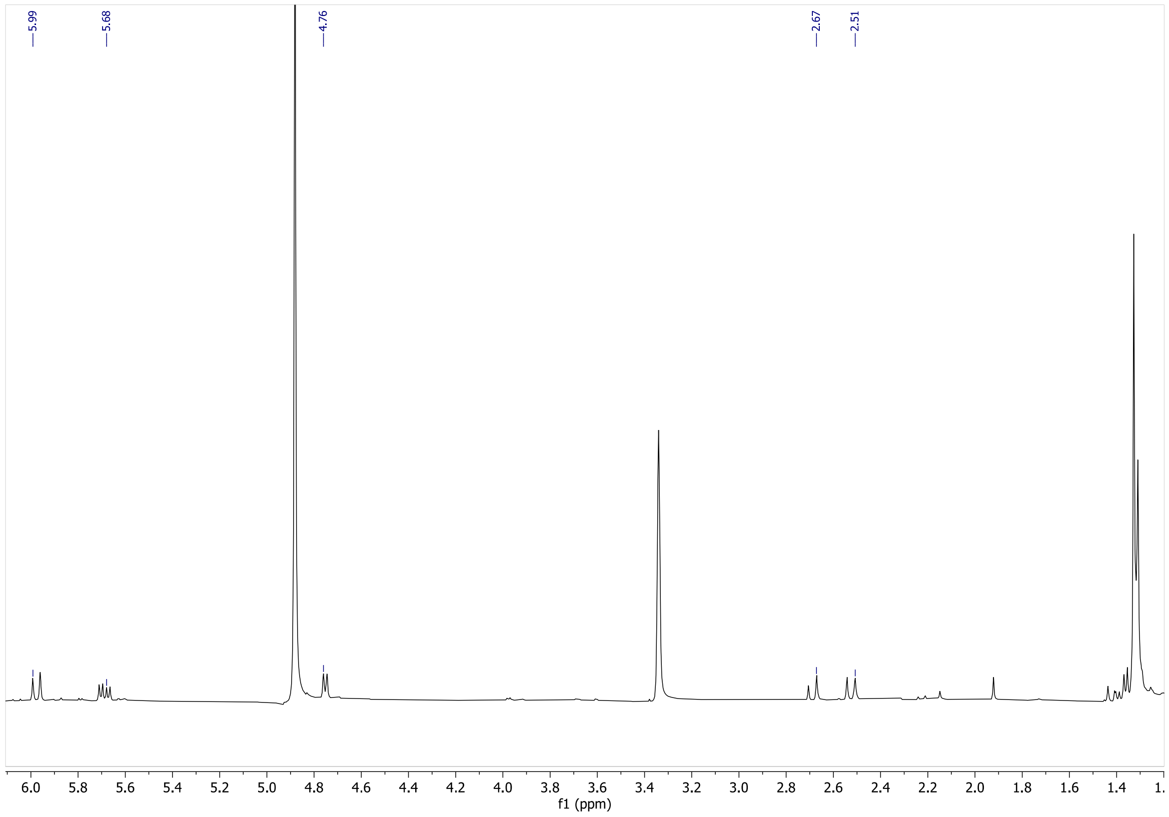

Figure S40. ^{13}C NMR spectrum of compound **7** (CD_3OD, 150 MHz)

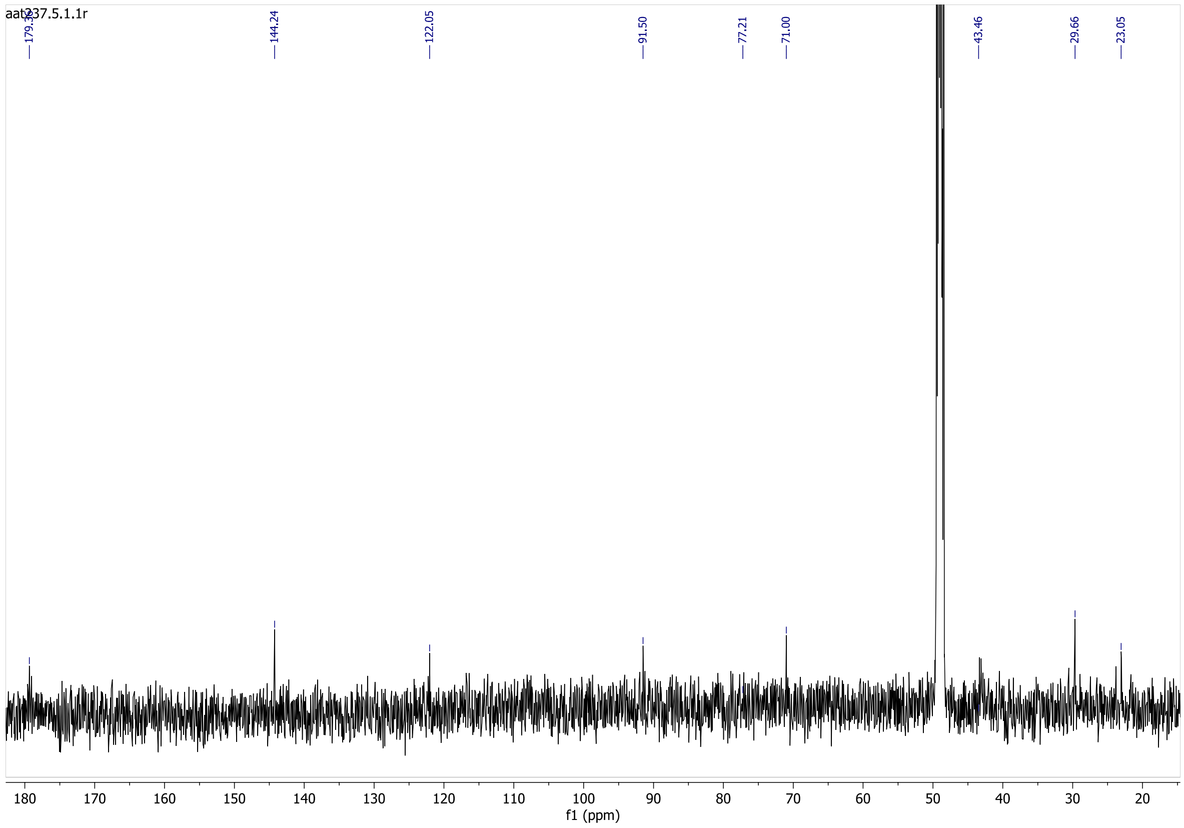

Figure S41. HSQC spectrum of compound **7** (CD_3OD, 600 MHz)

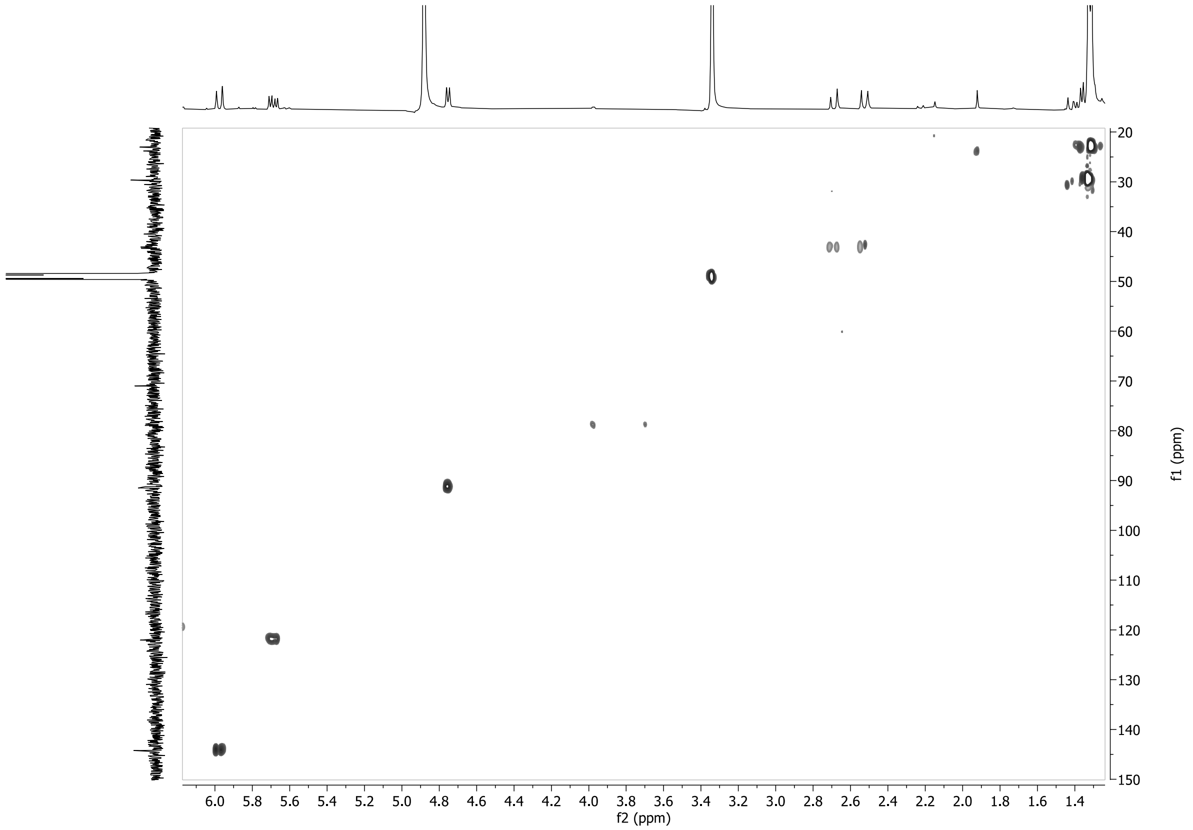

Figure S42. HMBC spectrum of compound **7** (CD_3OD, 600 MHz)

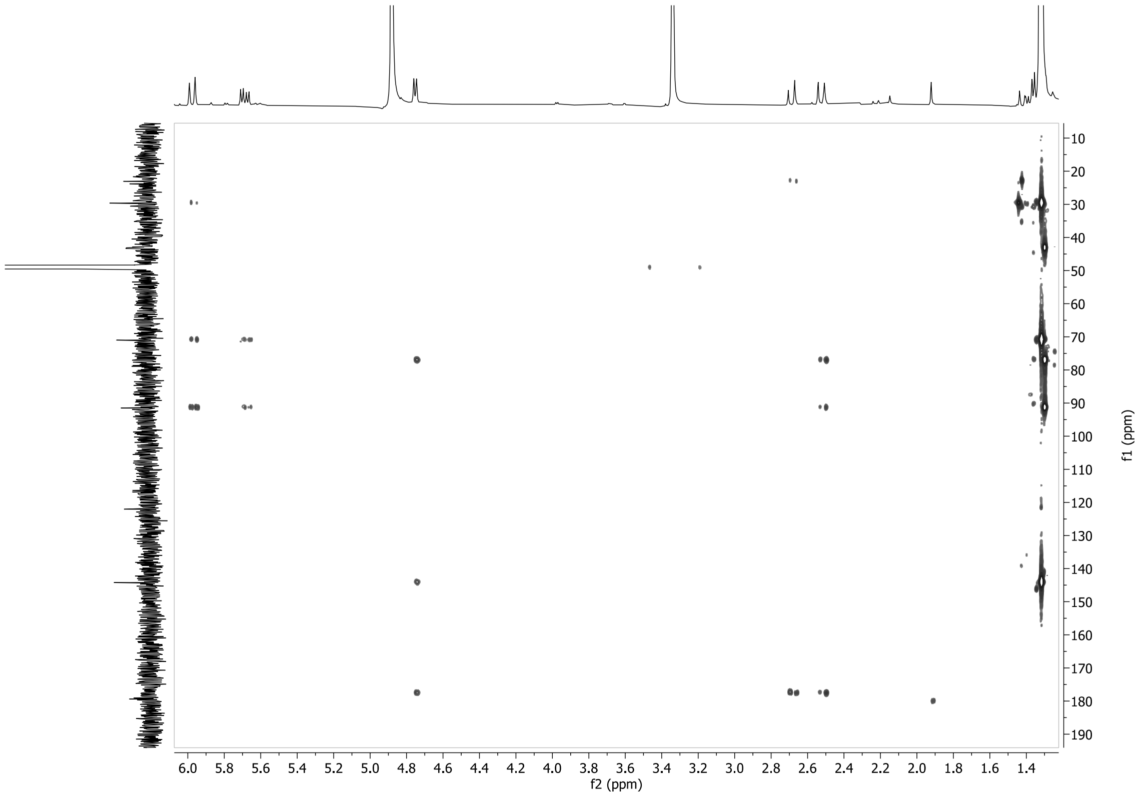

Figure S43. HRESIMS of compound **7**

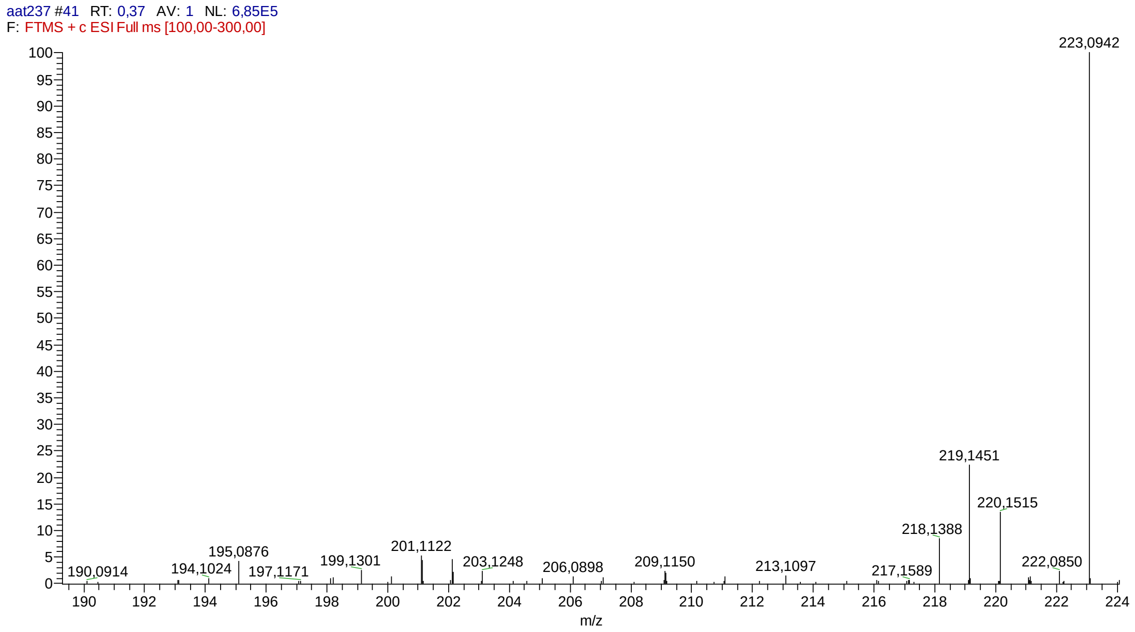

Table S1. Full MS and MS/MS data registered in negative ionization mode of compounds detected in the chloroform extract of *Ammoides atlantica* aerial parts

**Peak**[a]	**Compound**	t_R **(min)**	**HR-[M−H]⁻ (*m/z*)**	**HR-MS/MS product ions(*m/z*)**[b]	**Mol. Form.**	**Error (ppm)**
**a**	Carboxylic acid	1.7	155.0345	**111.04**	$C_7H_8O_4$	3.77
**7**	Dihydro-3-hydroxy-3-methyl-5-(3-hydroxy-3-methyl-1-buten-1-yl)-2(*3H*)-furanone	2.2	199.0972<br>*245.1030<br>**235.0741	***199.10**, 180.99, 153.09	$C_{10}H_{16}O_4$	3.74
**b**	Unknown	2.5	219.8452<br>*265.0719<br>**255.8223	*247.06, 221.08, 177.09, **159.08**		
**c**	Isomer of **1**	3.2	353.1243<br>*389.1011<br>**399.1297	**311.11**, 293.10	$C_{17}H_{22}O_8$	4.43
**5**	1,3,13-Trihydroxy-5-acetoxy-eudesma-4(15),7(11)-dien-12-oic acid	3.4	339.1440	293.10, 275.09, **231.10**, 203.11	$C_{17}H_{24}O_7$	2.65
**2**	1,4,9,10-Tetrahydroxy-5α,7α-H-guaia-2(3),11(13)-dien-12,6α-olide	3.7	295.1188<br>*341.1241<br>**331.0955	*295.12, 277.10, 233.12, 215.11	$C_{15}H_{20}O_6$	4.56
**d**	Dihydroxy-dodecadienoic acid	3.9	227.1287	209.12, 191.11	$C_{12}H_{20}O_4$	4.11
**1**	1,2,10-Trihydroxy-3,4-epoxy-9-acetoxy-5α,7α-H-guaia11(13)-en-12,6α-olide	4.0	353.1243<br>*399.1297<br>**389.1011	334.81, **293.10**, 275.09, 257.08	$C_{17}H_{22}O_8$	3.84
**8**, **9**	*epi*-Tanaphilin, *seco*-tanapartholide B	4.3	277.1080<br>*323.1135<br>**313.0849	259.10, 215.11, **197.10**	$C_{15}H_{18}O_5$	3.54
**10, 11**	9α-Acetoxyartecanin, apressin	5.1	335.1139<br>*381.1193<br>**371.0904	291.12, 275.09, 255.10, **213.09**, 195.08	$C_{17}H_{20}O_7$	3.97
**e**	Hydroxy-decatrienoic acid	5.2	181.0866	**137.10**, 119.09	$C_{10}H_{14}O_3$	3.53
**f**	Isomer of **10** and **11**	5.5	335.1138<br>*381.1193<br>**371.0904	275.09, 257.08, **231.10,** 213.09, 195.08	$C_{17}H_{20}O_7$	4.24
**3**	2-Oxo-6-hydroxy-8-acetoxyguai-1(10),3(4)-dien-12-oic acid.	5.7	321.1345<br>*367.1399<br>**357.1112	261.11, 243.10, 217.12, **59.01**	$C_{17}H_{22}O_6$	3.91
**13**	1β,3β-Dihydroxy-13-acetoxy-eudesma-4(15),7(11)-dien-12,6α-olide	6.0	319.1190<br>*365.1242	*292.89, **259.10**, 231.10	$C_{17}H_{20}O_6$	4.21
**6**	2,5,10-Trihydroxy-bisabol-3,12-diene	6.2	253.1806<br>*299.1865<br>**289.1578	*252.90, 234.88, 202.91, 174.91	$C_{15}H_{26}O_3$	3.27
**4**	1,5-Epoxy-guaian-10,11-diol	6.3	253.1811<br>*299.1865<br>**289.1578	*252.90, 234.88, 174.91	$C_{15}H_{26}O_3$	4.97
**12**	3α-Chloro-9α-acetoxy-4β,10α-dihydroxy-1β,2β-epoxy-5α,7αH-guai-11(13)-en-12,6α-olide	6.5	371.0903<br>*417.0960<br>**407.0671	**363.22, 344.81, **274.89**	$C_{17}H_{21}O_7Cl$	2.86

**g**	Hydroxylated sesquiterpene	7.2	267.1603 *313.1659 **303.1369	249.15, 223.17, 205.16, 193.16	$C_{15}H_{24}O_4$	4.36
**h, i**	Sesquiterpenes isomers	8.6, 8.7	377.1607 *423.1663 **413.1374	257.08, 231.10, 213.09, 195.08, 171.08, 143.05, 123.04, 93.03	$C_{20}H_{26}O_7$	3.54
**j**	Sesquiterpene dimer	9.1	523.2339 *569.2394 **559.2106	505.22, 479.24, **461.23**, 443.22, 233.11, 215.11, 145.06	$C_{30}H_{36}O_8$	2.13
**k**	Dimethoxylated flavonoid	9.2	329.0667	**314.04**, 299.02, 271.02	$C_{17}H_{14}O_8$	3.71
**14**	Gossypetin 3,7,3'-trimethyl ether	9.6	359.0773	344.05, 329.03, 314.00, 301.04, 286.01	$C_{18}H_{16}O_8$	3.22
**l**	Dimethoxylated flavonoid	12.0	313.0719	298.05, 283.02, 255.03	$C_{17}H_{14}O_6$	3.66
**15**	Tanetin	12.3	343.0821	328.06, 313.04, 285.04,	$C_{18}H_{16}O_7$	2.42

[a] Peaks indicated by numbers were identified by comparison with reference standards; peaks indicated by letters were tentatively identified based on MS data. [b]Product ions obtained by fragmentation of deprotonated molecular ions, except for data marked with asterisk; base ion peaks are shown in bold. *Formate adducts and their product ions. ** Chloride adducts and their product ions.

Table S2. Quantitative amounts (g/100 g ± SD of DW) of constituents isolated from *Ammoides atlantica* aerial parts.

Compound	Amount (g/100 g DW ± SD)
Sesquiterpenoids	
**1**	0.18 ± 0.01
**3**	0.04 ± 0.02
**8+9**	0.95 ± 0.08
**10+11**	1.33 ± 0.06
**12**	0.05 ± 0.04
Flavonoids	
**14**	0.14 ± 0.04
**15**	0.48 ± 0.05
Total sesquiterpenoids	2.55 ± 0.21
Total flavonoids	0.62 ± 0.09
Total compounds	3.17 ± 0.29

DW: dry weight; SD standard deviation.

Table S3. ^{13}C experimental and calculated NMR chemical shifts for **1**, with $^{a}|\Delta\delta|(^{13}C)$ and ^{b}MAE values.

Position	δ_{calc} (^{13}C), ppm<br>**1p**<br>1*S**,2*S**,3*R**,4*S**,9*S**,10*S**	δ_{exp} (^{13}C), ppm<br>**experimental**	$|\Delta\delta|$ (^{13}C), ppm[a]<br>**1p**<br>1*S**,2*S**,3*R**,4*S**,9*S**,10*S**
1	91.70	86.70	91.70
2	77.73	78.10	77.73
3	67.56	61.60	67.56
4	71.15	72.00	71.15
5	51.20	56.60	51.20
6	79.95	79.60	79.95
7	43.82	42.90	43.82
8	31.13	30.30	31.13
9	73.83	80.00	73.83
10	75.76	71.90	75.76
11	141.23	139.00	141.23
12	170.09	170.10	170.09
13	125.41	119.00	125.41
14	28.75	22.70	28.75
15	18.27	22.90	18.27
16	21.50	20.00	21.50
17	170.86	169.90	170.86
**MAE[b]**			**3.0**

[a] **$|\Delta\delta|(^{13}C)$** = $|\delta_{exp} - \delta_{calc}|$ (^{13}C), ppm: absolute differences for experimental versus calculated ^{13}C NMR chemical shifts; [b] **MAE** = $\Sigma[|(\delta_{exp} - \delta_{calcd})|]/n$, summation through n of the absolute error values (difference of the absolute values between corresponding experimental and ^{13}C chemical shifts), normalized to the number of the chemical shifts.

Table S4. ^{1}H experimental and calculated NMR chemical shifts for **1**, with $^a|\Delta\delta|(^1H)$ and ^{b}MAE values.

	δcalc (^{1}H), ppm	δ_{exp} (^{1}H), ppm	|Δδ| (^{1}H), ppm^a
	**1p**	**experimental**	**1p**
**Position**	1*S**,2*S**,3*R**,4*S**,9*S**,10*S**		1*S**,2*S**,3*R**,4*S**,9*S**,10*S**
2	3.84	3.69	0.15
3	3.61	3.62	0.01
5	2.29	2.39	0.10
6	4.50	4.61	0.11
7	2.90	3.15	0.25
8a	2.41	2.43	0.02
8b	1.87	1.85	0.02
9	5.36	5.11	0.25
13a	6.23	6.16	0.07
13b	5.52	5.60	0.08
14	1.39	1.38	0.01
15	1.54	1.36	0.18
16	2.00	2.14	0.14
**MAE^b**			**0.11**

^{a} **$|\Delta\delta|(^1H)$** = |δexp –δcalc| (^{1}H), ppm: absolute differences for experimental versus calculated ^{1}H NMR chemical shifts; ^{b} **MAE** = $\Sigma[|(\delta_{exp} - \delta_{calcd})|]/n$, summation through n of the absolute error values (difference of the absolute values between corresponding experimental and ^{1}H chemical shifts), normalized to the number of the chemical shifts.

Table S5. ^{13}C experimental and calculated NMR chemical shifts for **2**, with $^a|\Delta\delta|(^{13}C)$ and ^{b}MAE values.

	δ_{calc} (^{13}C), ppm	δ_{exp} (^{13}C), ppm	|Δδ| (^{13}C), ppm^a
	**2i**	**experimental**	**2i**
**Position**	1*S**,4*R**,9*R**,10*R**		1*S**,4*R**,9*R**,10*R**
1	92.69	93.50	0.81
2	137.37	134.30	3.07
3	144.23	140.00	4.23
4	83.63	82.70	0.93
5	63.93	67.20	3.27
6	80.75	83.00	2.25
7	40.48	39.10	1.38
8	34.61	34.50	0.11
9	78.67	80.60	1.93
10	75.51	77.00	1.49
11	141.39	140.80	0.59
12	170.03	172.10	2.07
13	127.20	120.30	6.90
14	23.63	22.80	0.83
15	23.61	22.00	1.61
**MAE^b**			**2.10**

^{a} **$|\Delta\delta|(^{13}C)$** = $|\delta_{exp} - \delta_{calc}|$ (^{13}C), ppm: absolute differences for experimental versus calculated ^{13}C NMR chemical shifts; ^{b} **MAE** = $\Sigma[|(\delta_{exp} - \delta_{calcd})|]/n$, summation through n of the absolute error values (difference of the absolute values between corresponding experimental and ^{13}C chemical shifts), normalized to the number of the chemical shifts.

Table S6. ^{1}H experimental and calculated NMR chemical shifts for **2**, with $^{a}|\Delta\delta|(^{1}H)$ and ^{b}MAE values.

	δcalc (^{1}H), ppm	δ_{exp} (^{1}H), ppm	$|\Delta\delta|$ (^{1}H), ppm^{a}
	**2i**	**experimental**	**2i**
**Position**	1*S**,4*R**,9*R**,10*R**		1*S**,4*R**,9*R**,10*R**
2	5.86	5.82	0.04
3	5.89	5.90	0.01
5	2.49	2.72	0.23
6	4.34	4.57	0.23
7	3.10	3.39	0.29
8a	2.40	2.34	0.06
8b	1.81	1.93	0.12
9	3.80	3.99	0.19
13a	6.35	6.22	0.13
13b	5.71	5.68	0.03
14	0.98	1.02	0.04
15	1.34	1.42	0.08
**MAE^{b}**			**0.12**

^{a} $\mathbf{|\Delta\delta|(^{1}H)}$ = |δexp –δcalc| (^{1}H), ppm: absolute differences for experimental versus calculated ^{1}H NMR chemical shifts; ^{b} **MAE** = $\Sigma[|(\delta_{exp} - \delta_{calcd})|]/n$, summation through n of the absolute error values (difference of the absolute values between corresponding experimental and ^{1}H chemical shifts), normalized to the number of the chemical shifts.

Table S7. ^{13}C experimental and calculated NMR chemical shifts for **6**, with $^{a}|\Delta\delta|(^{13}C)$ and ^{b}MAE values.

	δ_{calc} (^{13}C), ppm	δ_{exp} (^{13}C), ppm	$|\Delta\delta|$ (^{13}C), ppm^{a}
	**6d**	**experimental**	**6d**
**Position**	2*S**,5*R**,6*R**,7*S**,10*S**		2*S**,5*R**,6*R**,7*S**,10*S**
1	30.62	31.10	0.48
2	67.69	67.00	0.69
3	142.27	135.90	6.37
4	134.44	131.60	2.84
5	68.68	71.00	2.32
6	42.89	42.10	0.79
7	32.22	33.10	0.88
8	32.45	33.60	1.15
9	34.91	30.60	4.31
10	75.03	78.30	3.27
11	151.30	147.60	3.69
12	113.90	111.70	2.20
13	19.75	18.30	1.45
14	15.42	15.30	0.12
15	22.47	20.90	1.57
**MAE^{b}**			**2.14**

^{a} $\mathbf{|\Delta\delta|(^{13}C)}$ = $|\delta_{exp} - \delta_{calc}|$ (^{13}C), ppm: absolute differences for experimental versus calculated ^{13}C NMR chemical shifts; ^{b} **MAE** = $\Sigma[|(\delta_{exp} - \delta_{calcd})|]/n$, summation through n of the absolute error values (difference of the absolute values between corresponding experimental and ^{13}C chemical shifts), normalized to the number of the chemical shifts.

Table S8. ^{1}H experimental and calculated NMR chemical shifts for **6**, with $^a|\Delta\delta|(^1H)$ and ^{b}MAE values.

	δcalc (^{1}H), ppm	δ_{exp} (^{1}H), ppm	$|\Delta\delta|$ (^{1}H), ppm^a
	**6d**	**experimental**	**6d**
**Position**	2*S**,5*R**,6*R**,7*S**,10*S**		2*S**,5*R**,6*R**,7*S**,10*S**
1a	1.68	1.72	0.04
1b	1.41	1.33	0.08
2	3.79	3.96	0.17
4	5.50	5.52	0.02
5	3.99	3.89	0.10
6	1.22	1.75	0.53
7	2.17	2.01	0.16
8a	1.54	1.41	0.13
8b	1.19	1.24	0.05
9a	1.55	1.62	0.07
9b	1.49	1.62	0.13
10	3.99	4.03	0.04
12a	4.85	4.94	0.09
12b	4.75	4.83	0.08
13	1.74	1.74	0.00
14	0.77	0.85	0.08
15	1.77	1.81	0.04
**MAE^b**			**0.11**

^{a} **$|\Delta\delta|(^1H)$** = |δexp –δcalc| (^{1}H), ppm: absolute differences for experimental versus calculated ^{1}H NMR chemical shifts; ^{b} **MAE** = $\Sigma[|(\delta_{exp} - \delta_{calcd})|]/n$, summation through n of the absolute error values (difference of the absolute values between corresponding experimental and ^{1}H chemical shifts), normalized to the number of the chemical shifts.

Table S9. ^{13}C experimental and calculated NMR chemical shifts for **7**, with $^a|\Delta\delta|(^{13}C)$ and ^{b}MAE values.

	δ_{calc} (^{13}C), ppm	δ_{exp} (^{13}C), ppm	$|\Delta\delta|$ (^{13}C), ppm^a
	**7a**	**experimental**	**7a**
**Position**	3*S**,4*R**		3*S**,4*R**
1	174.77	177.30	2.53
2	43.00	43.00	0.00
3	76.84	77.00	0.16
4	88.74	90.80	2.06
5	122.48	121.70	0.78
6	148.14	143.30	4.83
7	70.50	70.70	0.20
8	29.57	29.90	0.33
9	29.11	29.90	0.79
10	22.95	23.00	0.05
**MAE^b**			**1.17**

^{a} **$|\Delta\delta|(^{13}C)$** = $|\delta_{exp} - \delta_{calc}|$ (^{13}C), ppm: absolute differences for experimental versus calculated ^{13}C NMR chemical shifts; ^{b} **MAE** = $\Sigma[|(\delta_{exp} - \delta_{calcd})|]/n$, summation through n of the absolute error values (difference of the absolute values between corresponding experimental and ^{13}C chemical shifts), normalized to the number of the chemical shifts.

Table S10. ^{1}H experimental and calculated NMR chemical shifts for **7**, with [a]|Δδ|(^{1}H) and [b]MAE values.

	δcalc (^{1}H), ppm	δ_{exp} (^{1}H), ppm	|Δδ| (^{1}H), ppm[a]
**Position**	**7a** 3*S**,4*R**	**experimental**	**7a** 3*S**,4*R**
1a	2.43	2.53	0.10
1b	2.61	2.69	0.08
2	4.72	4.74	0.02
4	5.74	5.67	0.07
5	6.10	5.97	0.13
6	1.24	1.32	0.08
7	1.23	1.32	0.09
8a	1.20	1.31	0.11
**MAE[b]**			**0.08**

[a] **|Δδ|(^{1}H)** = |δexp –δcalc| (^{1}H), ppm: absolute differences for experimental versus calculated ^{1}H NMR chemical shifts; [b] **MAE** = $\Sigma[|(\delta_{exp} - \delta_{calcd})|]/n$, summation through n of the absolute error values (difference of the absolute values between corresponding experimental and ^{1}H chemical shifts), normalized to the number of the chemical shifts.
